# Layer Number Dependence of Chirality and Spin Polarized Lifetime in Chiral 2D Halide Perovskites

**DOI:** 10.1021/jacs.5c16029

**Published:** 2025-11-19

**Authors:** Shripathi Ramakrishnan, Yifan Dong, Yi Xie, Jacob L. Shelton, Matthew P. Hautzinger, Duong Nguyen Minh, Margherita Taddei, Xiaoyu Zhang, Yugang Zhang, David B. Mitzi, Md Azimul Haque, Jeffrey L. Blackburn, Qiuming Yu, Matthew C. Beard, Joseph M. Luther

**Affiliations:** + 53405 National Renewable Energy Laboratory, Golden, Colorado 80401, United States; ‡ Robert Frederick Smith School of Chemical and Biomolecular Engineering, 5922 Cornell University, Ithaca, New York 14853, United States; § Department of Chemistry, 8790 University of California, Riverside, California 92521, United States; ∥ Thomas Lord Department of Mechanical Engineering and Materials Science, 3065 Duke University, Durham, North Carolina 27708, United States; ⊥ Department of Chemistry, 3065 Duke University, Durham, North Carolina 27708, United States; # Center for Functional Nanomaterials, 8099 Brookhaven National Laboratory, Upton, New York 11973-5000, United States

## Abstract

Chiral metal halide perovskite semiconductors (CMHS) are fascinating semiconductors with unique chiroptical properties and spin-polarized charge transport. Achieving long spin lifetimes and high carrier mobility concurrently is essential to realize the true potential of CMHS in manipulating charge, spin, and light. While conventional monolayer *n* = 1 CMHS possess appreciable anisotropy factors of circular dichroism (g_CD_) and photoluminescence (g_lum_), imparting chirality to quasi-2D CMHS (*n* > 1) with enhanced carrier mobilities is underexplored. Herein, we systematically investigate the layer number (*n*-value) dependence and emergent trade-offs in chiroptical properties, spin-relaxation times, and carrier mobilities in chiral quasi-2D (R/S-MPEA)_2_MA_n‑1_Pb_n_I_3n+1_ single crystals and thin films (R/S-MPEA: R/S-β-methylphenylethylammonium; MA: methylammonium; *n* = 1–3). Films with *n* = 2 exhibited the highest *g*_CD_ of 8 × 10^–3^, an order of magnitude larger than their *n* = 1 and *n* = 3 counterparts. On the other hand, *n* = 3 films demonstrated enhanced spin lifetimes up to 15 ps along with increased carrier mobility up to 11.6 cm^2^ V^–1^ s^–1^. As a result, photodiode-type photodetectors based on *n* = 3 CMHS reveal high specific detectivity and superior discrimination of circularly polarized light, outperforming *n* = 1 and 2. These findings highlight the potential of quasi-2D CMHS as tunable, high-performance platforms with longer spin lifetime and diffusion length, enabling new functionalities.

## Graphical abstract

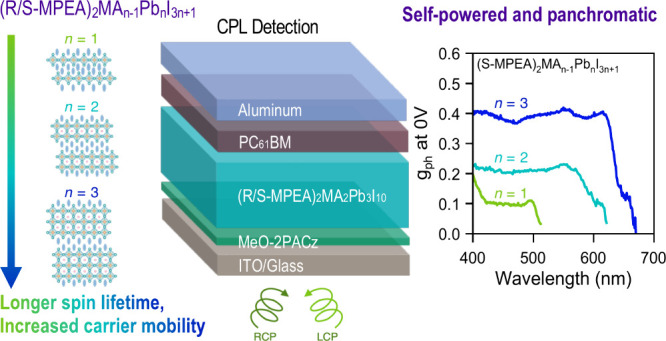

## Introduction

Two-dimensional metal-halide perovskites (2D-MHPs) are a class of structurally diverse semiconductors that offer opportunities for future energy-efficient computing, communications, sensing, and energy conversion technologies.^1,2^ 2D-MHPs are denoted by the formula A′_2_A_*n*‑1_Pb_*n*_X_3*n*+1_ and are comprised of a bulky organic monovalent cation A′ that acts as a spacer between [A_n‑1_Pb_n_X_3n+1_]^2–^ perovskite sheets (A = small monovalent cation, X = halide).^3,4^ The bulky A′ spacers impose a strong quantum and dielectric confinement effect, giving rise to significant exciton binding energies (E_b_), spectral tunability and adjustable cross-layer energy landscape.^3,5–9^ In particular, the incorporation of chiral organic spacers offers a pathway to induce structural chirality in the inorganic frameworks, breaking the inversion symmetry, enhancing spin-splitting phenomena (e.g., Rashba-Dresselhaus) and modifying the electronic structure and optical properties.^10,11^ The intrinsic chirality of the resulting chiral metal-halide perovskite semiconductors (CMHS) has enabled interesting and beneficial phenomena such as circularly polarized light detection and emission, spin-polarized charge transport, bulk photovoltaic effects and spin-dependent photogalvanic response.^12–17^

Recent reports have focused on enhancing the chiroptical properties of *n* = 1 CMHS by rational chiral spacer selection, spacer mixing and additive incorporation, with a goal of investigating the relationship between average bond angle distortion, bond-angle disparities, hydrogen bond asymmetry and circular dichroism anisotropy (g_CD_) factors.^10,18–21^ However, highly distorted lattices are also prone to wave function localization, defects and dangling bonds that can impede carrier transport, limit radiative efficiency and degrade structural stability.^22–28^ In terms of charge transport, *n* = 1 CMHS have low electrical conductivities (c.a. 10^–5^ to 10^–7^ S cm^–1^) and carrier mobilities (10^–2^ to 10^–3^ cm^2^ V^–1^ s^–1^), which present potential difficulties in electrically generating and detecting spin-polarized currents.^29–32^ The carrier mobility is also anisotropic across different planes in the *n* = 1 CMHS, with the mobility along the in-plane direction (along the inorganic octahedra) exceeding the out-of-plane direction (along the stacking axis, and across the organic spacers) by over an order of magnitude.^33^ Most critically, the spin lifetime (the time a carrier retains its optically oriented spin polarization before randomizing) is a crucial consideration toward realizing efficient spin functionalities.^5,34^

In this regard, quasi-2D halide perovskites (*i.e.*, *n* > 1) have the potential to alleviate these issues by virtue of their reduced bandgaps, reduced phonon scattering at the organic–inorganic interfaces, low exciton binding energies and correspondingly larger charge carrier mobilities and conductivities.^35–37^ It is expected that inclusion of the small A-site cation may reduce the g_CD_ as a result of reduced octahedral distortion and fraction of chiral spacers. In exchange, the spin lifetimes of achiral quasi-2D perovskites take advantage of the Rashba splitting, reduced exciton binding energy, and reduced phonon scattering to exhibit elongated spin lifetime and carrier mobility. As such, exploring the trade-offs between chiroptical activity and spin transport properties by expanding the chemical space of CMHS into quasi-2D CMHS with larger layer numbers could be very valuable. However, quasi-2D CMHS can often lead to the formation of phase segregated mixtures of chiral *n* = 1 and achiral 3D-MHPs.^38–41^ Indeed studying polydisperse films composed of multiple phases as opposed to a narrow phase distribution with a homogeneous energy landscape impedes a systematic understanding of the effect of layer number *n* on the chiroptical and spin properties of CMHS.

In this work, we systematically investigate and compare the chiroptical properties and spin lifetimes in a series of CMHS with varying layer number *n*. We successfully designed phase-pure crystals based on the formula (R/S-MPEA)_2_MA_n‑1_Pb_n_I_3n+1_ (R/S-MPEA = R/S-β-methylphenylethylammonium, *n* = 1 – 3) as well as thin films dominated by nearly the same *n* as the parent crystal to examine the chiroptical properties of a specific *n*. We find that for increasing layer number, the quasi-2D CMHS adopt a vertical orientation with the inorganic layers aligned perpendicular to the substrate as opposed to the horizontal orientation for *n* = 1 CMHS, offering improved carrier transport in vertical architectures. Circular dichroism measurements revealed that the *n* = 2 films exhibit a maximum g_CD_ of 8 × 10^–3^, which is an order of magnitude larger than the respective *n* = 1 and 3 films. On the other hand, the *n* = 3 films exhibit a relatively high carrier mobility of 11.6 cm^2^ V^–1^ s^–1^ compared to *n* = 1 (1.5 cm^2^ V^–1^ s^–1^) and *n* = 2 (5.2 cm^2^ V^–1^ s^–1^) and an enhanced spin lifetime up to 15 ps. Photodiode-type circularly polarized light detectors based on *n* = 3 resulted in enhanced distinguishability compared to the *n* = 1 and 2 based photodetectors despite the low g_CD_. Finally, we turned to modeling of self-powered photodetectors under CPL illumination from the perspective of photogeneration and transport of spin-polarized carriers to highlight the critical role of managing spin relaxation and spin-specific carrier mobility in structures that harness the spin degree of freedom.

## Results and Discussion

The selection of a suitable chiral spacer molecule has a crucial impact on the formation of CMHS with higher layer numbers. The chiral spacer influences the crystal configuration of the inorganic octahedra on many levels, such as the organic–inorganic interactions, intra- and interoctahedral distortions, structural symmetry, and interlayer distance.^10^ We observed that the commonly studied chiral spacers (R/S-methylbenzylammonium R/S-MBA^+^, R/S-1,1-naphythyl­ethylammonium R/S-NEA^+^) do not form quasi-2D CMHS and instead, for R/S-MBA^+^, phase segregate into a mixture of chiral (R/S-MBA)_2_PbI_4_ and MAPbI_3_ (Figures S1 and S2, supplementary note 1). This is possibly due to significant lattice mismatch between the *n* = 1 and 3D MAPbI_3_ and FAPbI_3_ structures, causing internal lattice strain.^42^ We hypothesized that the longer tethering group in R/S-MPEA, compared with those in R/S-MBA and R/S-NEA, enhances cation flexibility and thereby facilitates its accommodation within the inorganic framework. Therefore, we turned to R/S-MPEA^+^ on the basis of its structural similarity to its achiral counterpart, phenylethylammonium (PEA^+^), which has been used to successfully synthesize homologous 2D (PEA)_2_MA_n‑1_Pb_n_I_3n+1_ (*n* = 1–4).^43^ Phase-pure crystals with the formula (R/S-MPEA)_2_MA_n‑1_Pb_n_I_3n+1_ (Figure 1a) were synthesized via the slow-cooling of an off-stoichiometric mixture of R/S-MPEA, methylammonium iodide MAI and lead iodide PbI_2_ dissolved in hydroiodic acid.^3^ A full description of the growth is provided in the Supporting Information. We verified the presence of three inorganic layers in (R-MPEA)_2_MA_2_Pb_3_I_10_ through single-crystal X-ray diffraction, as shown in Figure S3a-b. Comparing the simulated XRD with that of the CMHS crystals revealed good matching with peaks corresponding to the stacking direction and Pb–Pb distance (Figure S3c).

**Figure 1:**
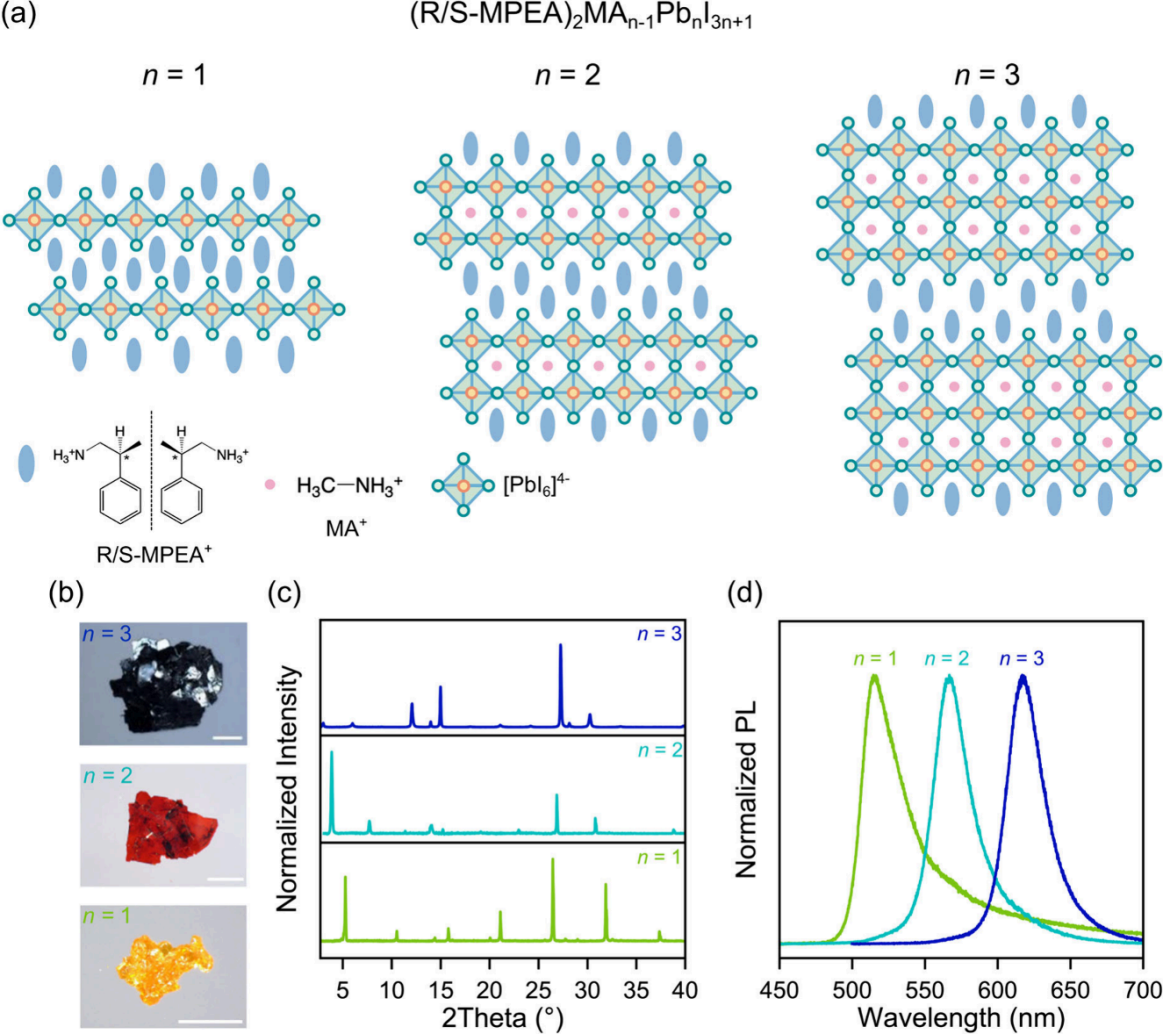
(a) Schematic representation of the layered crystal structure, (b) optical image (scale bars are 1 mm), (c) powder X-ray diffraction pattern and (d) photoluminescence spectra of *n* = 1–3 (R-MPEA)_2_MA_n‑1_Pb_n_I_3n+1_ crystals.

As shown in Figure 1b, the color of the (S-MPEA)_2_MA_n‑1_Pb_n_I_3n+1_ crystals varies from orange (*n* = 1), to red (*n* = 2) and black (*n* = 3). The powder X-ray diffraction (XRD) spectra featured equally spaced peaks corresponding to the stacking axis of 2D-MHPs (Figure 1c), with interlayer spacings of 16.76, 23.05, and 29.43 Å for *n* = 1 – 3 respectively (Figure S4a) consistent with the increasing layer number (i.e., increasing by around 6.3–6.4 Å, corresponding to approximately two Pb–I bond lengths, for each increment in *n*). The XRD patterns are similar to those of the previously synthesized (β-Me-PEA)_2_MA_n‑1_Pb_n_I_3n+1_ series in terms of peak position and distribution.^44^ The PL spectra (Figure 1d) of the single crystals are dominated by a single emission peak which bathochromically shifts with increasing *n* from 516 nm for *n* = 1 to 620 nm for *n* = 3 (Figure S4b), indicative of phase-purity of the synthesized single crystals. It should be noted that we succeeded in synthesizing *n* = 4 crystals in their phase-pure forms (Figure S5a-b). However, this series is not included in our study due to challenges in translating them into thin film architectures (Figure S5c).

To better understand the crystal structure and compare the effect of layer number *n* on the chiroptical properties, thin films were fabricated using the larger crystals as precursors. Multilayer CMHS films were fabricated by dispersing the parent (S-MPEA)_2_MA_n‑1_Pb_n_I_3n+1_ crystals of each *n* value in a weakly coordinating solvent mixture of acetonitrile and tetrahydrofuran and spin casting the mixture onto quartz substrates. This approach significantly reduces solvent intercalation into the dispersed crystals, preserving their initial structure and seeding the growth of thin films with nearly the same phase distribution of *n* as the parent crystals.^42^ The UV–vis spectra of the corresponding (S-MPEA)_2_MA_n‑1_Pb_n_I_3n+1_ films (Figure 2a) feature strong excitonic peaks corresponding to the same *n* value as the parent crystals and follow a general trend where the excitonic peak gets red-shifted in defined steps as the *n* increases, with the excitonic peak shifting from 501 nm for *n* = 1 to 551 nm for *n* = 2 and 605 nm for *n* = 3. The excitonic peaks also become less prominent as *n* increases, consistent with previous reports that the exciton binding energy, E_b_, reduces with increasing *n*.^43^ The PL spectra (Figure 2b) exhibit an analogous behavior, with the emission band edge red-shifting as a function of *n*.

**Figure 2:**
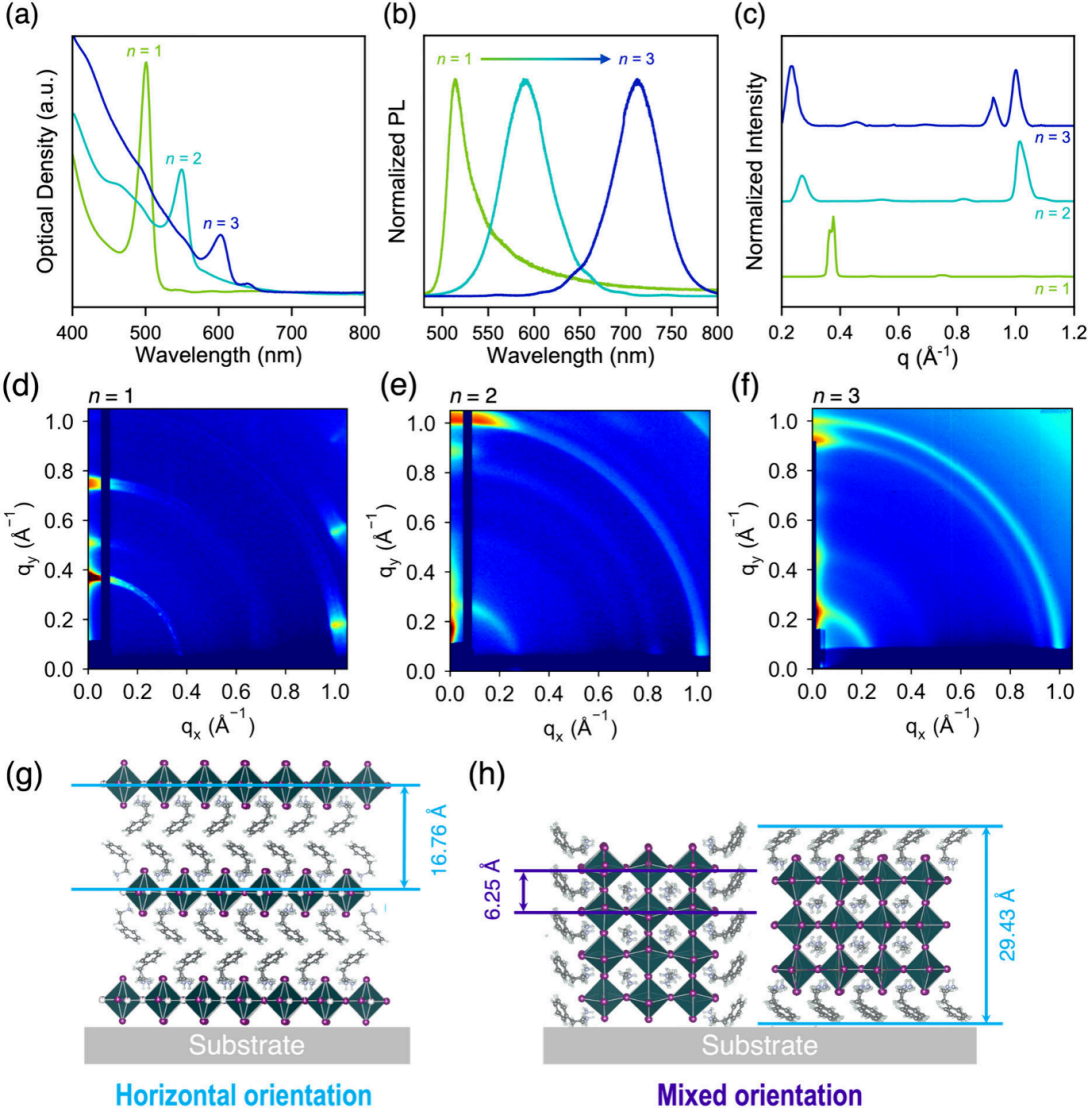
UV–vis (a), photoluminescence (b) and circularly averaged grazing-incidence wide-angle X-ray scattering (GIWAXS) spectra (c) of *n* = 1–3 (R-MPEA)_2_MA_n‑1_Pb_n_I_3n+1_ films. 2D GIWAXS maps of *n* = 1, *n* = 2 and *n* = 3 (R-MPEA)_2_MA_n‑1_Pb_n_I_3n+1_ films (d–f). Schematic representation of crystal orientations in *n* = 1 (g) and *n* = 2 – 3 (h) films based on peak intensity distribution of lower q and q = 1 Å^–1^ peaks.

Quasi-2D CMHS are expected to possess an increased propensity for growth with their inorganic layers perpendicular to the substrate.^45^ To gain insights into the crystal orientation of the prepared films, we performed grazing-incidence wide-angle scattering (GIWAXS) measurements at an incident angle of 0.5°. The circularly averaged linecuts (intensity vs q) and q_*x*_ vs q_*y*_ maps as a function of q are presented in Figure 2c-f, respectively. The *n* = 1 sample displays intense Bragg spots along the q_*y*_ axis at c.a. 0.38 Å^–1^ (d = 16.76 Å^–1^), corresponding to the stacking distance, and a faint spot along the q_*x*_ axis at 1.02 Å^–1^ (d = 6.15 Å^–1^), corresponding to the equatorial Pb–Pb distance. The *n* = 2 and 3 samples display rings at 0.27 Å^–1^ and 0.22 Å^–1^ (d = 23.29 and 28.54 Å^–1^ respectively) and rings at 1 Å^–1^ (d = 6.28 Å^–1^), with prominent intensity along the q_*y*_ axis. The observation of equally spaced peaks in the low-q region that correspond to the *d*-spacings along the stacking axes of the respective CMHS phases is in agreement with the composition indicated by the UV–vis spectra, confirming the formation of thin films dominated by the same *n* as the parent crystals. The current film processing strategy enables the realization of *n* = 1 – 3 films with the target *n* value of choice with high phase selectivity compared to previous works, for which nominal precursors did not produce the intended composition.^39,41^ To examine the orientational features in the *n* = 1–3 films, we integrated the region between 0.97 and 1.05 Å^–1^ to capture the variation of the peak intensity of the equatorial Pb–Pb distance as a function of the azimuthal angle (Figure S6a-c). Due to rotational isotropy around the substrate normal, the scattering is symmetric in the azimuthal angle. The distribution from 0° to 90° provides comprehensive information about the orientation of the crystallites, while patterns between 90° and 180° indicate symmetric diffractions. For the *n* = 1 film, the majority of the peak’s intensity is at c.a. 10° and 170°, suggesting that the inorganic framework of the *n* = 1 crystallites predominantly self-assemble with their inorganic frameworks parallel to the substrate, adopting a horizontal orientation (Figure 2g). On the other hand, while the majority of the peak is concentrated at 90° for *n* = 2 (and 3), there is significant intensity in the 0°–70° and 110°–180° regions as well.

Thus, the quasi-2D CMHS films adopt a mixture of orientations, with a preference for self-assembling with the [PbI_6_]^4–^ octahedral layers aligned perpendicular to the substrate (Figure 2h). Since the mobility of 2D-MHPs along the inorganic framework is almost 2 orders of magnitude larger as compared to across the organic layer, the quasi-2D CMHS have promising implications for the control over the anisotropic spin and charge carrier transport, compared to the conventional *n* = 1 systems wherein control over the layer orientation is more difficult.^33^ Importantly, spin transport parameters such as spin diffusion length are typically limited by the low carrier mobility along the out-of-plane direction in ‘vertical structures’, in which a perovskite film is sandwiched between two electrodes. Thus, managing crystal orientation in chiral perovskites may facilitate longer spin diffusion lengths by aligning the in-plane axis with the direction of applied field or spin injection in vertical heterostructures.

We compared the chiroptical activity of the *n* = 1–3 films by collecting circular dichroism (CD) spectra (Figure 3a-c), which exhibit several features of interest. Strong CD signals were observed for all three films, with derivative-like CD features (Cotton effect) near the excitonic resonance. This effect is a consequence of spin splitting via cross-coupling of Rashba-like and chiral/helical spin texture components.^46^ The *n* = 1 films exhibit a maximum CD of c.a. ± 11 mdeg, while the *n* = 2 films exhibit a larger CD of c.a. ± 85 mdeg and *n* = 3, ± 3 mdeg, for films with comparable thicknesses (c.a. 200–250 nm). Because of the enhancement in CD and reduction in optical density of the excitonic peak, the *g*_CD_ of *n* = 2 exhibits an order of magnitude increase to c.a. 8 × 10^–3^ (2.5 × 10^–3^ at the excitonic peak) compared to *n* = 1, which is c.a. 2 × 10^–4^, while that of *n* = 3 is c.a. 5 × 10^–4^.

**Figure 3:**
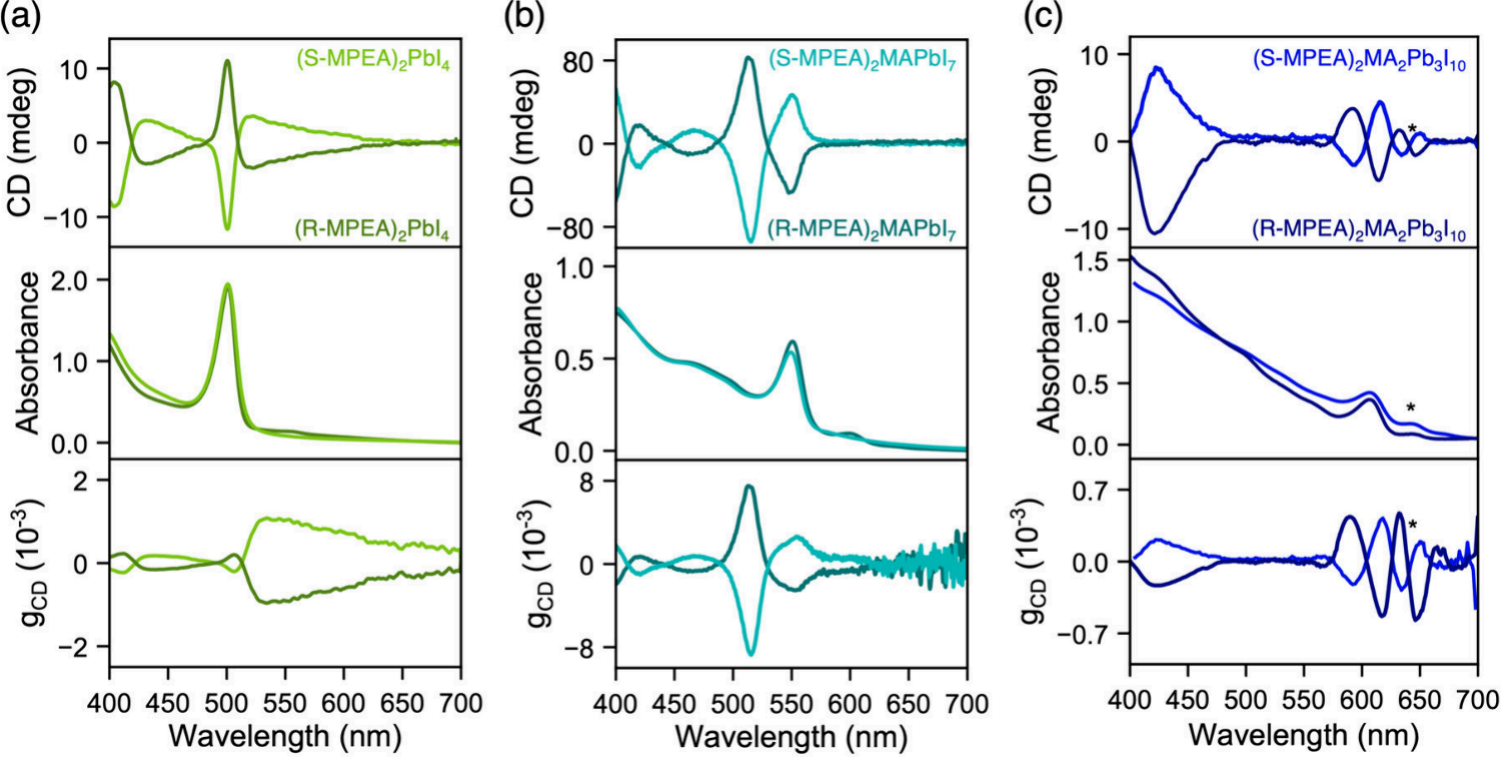
Circular dichroism (CD), UV–vis absorption and anisotropy factor of circular dichroism *g*_CD_ spectra of (a) *n* = 1, (b) *n* = 2, and (c) *n* = 3 CMHS films with the formula (R/S-MPEA)_2_MA_n‑1_Pb_n_I_3n+1_. The asterisk in (c) denotes the *n* = 4 impurity phase.

Importantly, the SEM images (Figure S7a-d) reveal a relatively flat morphology free of pinholes and texturing. We also checked for asymmetric linear-dichroism and linear-birefrigerance (LD-LB) effects by collecting CD spectra from the glass side (back) and film side (front). As shown in Figure S8a-c, the samples show very similar CD spectra under both configurations, confirming that the CD is likely intrinsic to the material and is not obscured by variations in morphology.^47^ The g_CD_ diminishes substantially when the layer number increases from 2 to 3. This could be due to a mixture of orientations in the *n* = 3 film, with different crystal facets possessing varying degrees of chiroptical activity depending on the spin textures prevailing along a crystallographic axis.^47^ Finally, a noticeable CD arises from the *n* = 4 impurity phase in Figure 3c (g_CD_ = 5 × 10^–4^) denoted by the asterisk, which is inevitably formed as a minority component during the growth of quasi-2D phases. Impressively, the chiroptical activity is preserved even up to four octahedra-thick CMHS. In conclusion, the chiroptical activity is nonmonotonic reaching a maximum at *n* = 2, and then diminishing for larger layer thicknesses.

We turned to time-resolved spectroscopy to characterize the spin lifetimes, carrier lifetimes and carrier mobilities as a function of layer number. We employed time-resolved circular dichroism (TRCD) to measure the spin lifetimes in thin films and single crystals. Figure S9 shows the band diagram for optical transitions in MHPs where the upper valence band and lower conduction band have |*J*, *m*_*J*_⟩ states of |1/2, ±1/2⟩. Under the circularly polarized pump, photons with an angular momentum of ±1 (for left or right circular polarization σ^+^/σ^–^) will selectively generate spin-polarized carriers following angular momentum conservation (i.e., Δ*m*_*J*_ = ±1). The decay of spin-polarized carriers can be probed as a function of time by measuring the photoinduced CD. This ellipsometry-based approach allows us to analyze the ellipticity change of the linearly polarized probe following spin-selective optical orientation, from which we can extract the lifetime of spin-polarized carriers. Detailed description of the TRCD method can be found in the SI.

Figure 4a shows the normalized TRCD kinetics for thin films with varying layer numbers from *n* = 1–3. A comparison between the TA and TRCD spectra can be found in Figure S10a-c. We initially performed fluence-dependent measurements and identified the lowest pump fluence in order to avoid nonlinear effects and many-body interactions (Figure S11a-c). All samples show decays that can be fitted empirically with a biexponential function. In the *n* = 1 sample, the slower component of the decay was found to be ∼5 ps. This slow component increases to 11 ps in the *n* = 2 sample and 15 ps in the *n* = 3 sample. In addition, we compared the spin lifetimes in thin films vs single crystals at similar excitation density to rule out any contribution from the *n* = 4 impurity, as the single crystals are of superior phase purity relative to thin films. Critically, single crystal and thin film show comparable spin lifetimes (Figure S12), confirming that the small portion of phase impurity does not have a significant impact on the measured spin lifetime.

**Figure 4:**
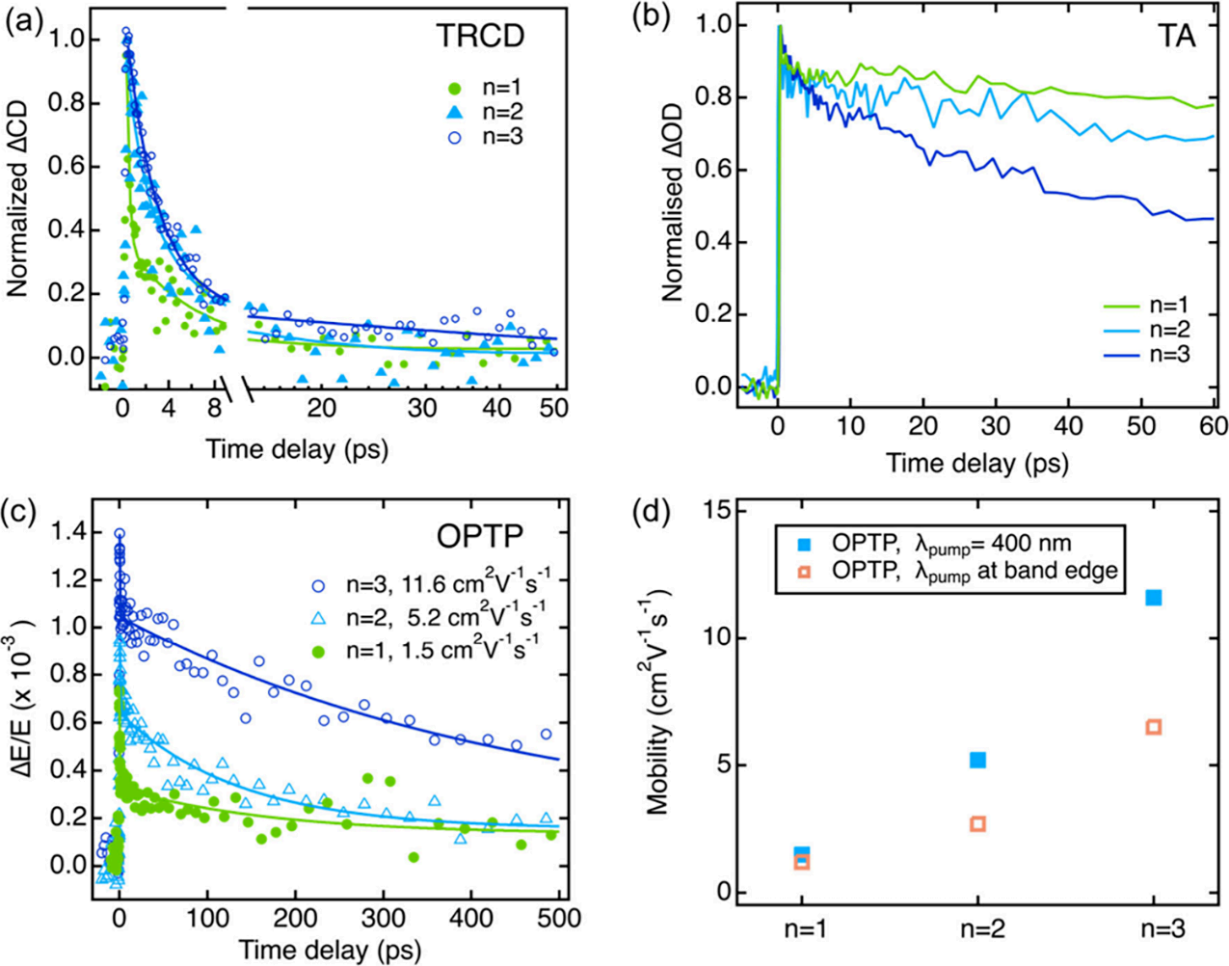
Ultrafast optical spectroscopy characterization for *n* = 1–3 thin films: (a) Normalized time-resolved circular dichroism (TRCD) kinetics. (b) Normalized transient absorption kinetics measured at excitation densities of 2 × 10^17^ cm^–3^ for *n* = 1, 3.1 × 10^17^ cm^–3^ for *n* = 2, and 3.6 × 10^17^ cm^–3^ for *n* = 3 films, respectively. (c) Optical pump THz probe (OPTP) kinetics excited at 400 nm. (d) Summary of mobility values extracted from OPTP measurements at 400 nm vs exciton-resonant excitations.

We also simultaneously measured transient absorption spectroscopy for the same films while running TRCD. As shown in Figure 4b, the carrier lifetimes in *n* = 1–3 films are much longer-lived than the spin lifetimes measured. Interestingly, the carrier lifetime decreases with increasing layer number, opposite to the spin lifetime. Thus, a larger proportion of the initial optically injected spin can be utilized or manipulated in the *n* = 3 phase, relative to *n* = 1 or 2, since the longer spin lifetime can preserve the spin more efficiently over the duration of the carrier lifetime.

Optical pump/THz probe (OPTP) spectroscopy was carried out to probe variations in charge carrier transport and elucidate the impact of layer number on carrier mobility in the *n* = 1–3 films. OPTP measurements provide noncontact characterization of short-range photoinduced charge transport, with picosecond resolution. Figure 4c shows the OPTP dynamics for *n* = 1–3 films, excited at 400 nm. OPTP measures the change in THz transmission, *ΔE*/*E*, upon photoexcitation and is propotional to the change in conductivity, *ΔE*/*E* = (Δσ·*z*)/((1+*n*_*s*_)*c*·*ε*_0_), where *z* is the film thickness, *n_s_* is the refractive index of the substrate, *c* is the speed of light, and *ε*_0_ is the permittivity of free space. The photoinduced change in conductivity is related the charge carrier mobility through *Δσ* = *N*_*abs*_·*f*·(μ_*e*_ + μ_*h*_), where *N*_*abs*_ is the absorbed photon fluence, μ_*e*/*h*_ is the electron/hole mobility, and *f* is the branching ratio between free charge carriers and excitions. To determine the nature of the charge carriers, OPTP was further carried out exciting thin films at the excitonic resonance where excitonic features dominate and a mixture of excitons and free carriers are photoinduced. As Figure S13 shows, exciting at the excitonic resonance does not show the fast decay but rather the kinetics resembles the slower component of the carrier decay under 400 nm excitation. Thus, we attribute the initial fast decay in Figure 4c to exciton formation kinetics. The THz probe is only sensitive to free carriers and with 400 nm excitation light only free carriers are produced and thus *f*∼1 and the measured, *ΔE*/*E*, is directly related to the free carrier mobility μ = (μ_*e*_ + μ_*h*_). At later times or when photoexciting resonant with exciton transition, *f* < 1, which reduces the photoinduced conductivity, *Δσ*. The rate of exciton formation will depend upon the exciton binding energy, carrier mobility, and carrier density. In this study we do not attempt to study the exciton formation dynamics and are only interested in the initial carrier mobility. We summarized the mobility for *n* = 1–3 films measured for both above-gap (400 nm) and resonant excitation in Figure 4d, which demonstrates a systematic increase in carrier mobility with increasing *n* for both excitation conditions. The difference between resonant and 400 nm excitations follows the trend in exciton binding energy; at 400 nm mostly free carriers are produced, so at early times this reflects the free-carrier mobility. The apparent lower mobility at longer times or when exciting under resonant conditions results because some of the excitation produces excitons which do not absorb THz radiation. Under 400 nm excitation, where free carriers respond, we found a 3-fold increase in mobility going from *n* = 1 to *n* = 2. The mobility further increased from 5.2 cm^2^ V^–1^ s^–1^ in the *n* = 2 to 11.6 cm^2^ V^–1^ s^–1^ in the *n* = 3 sample. Thus, increasing layer number presents an efficient way to enhance both the spin lifetime and carrier transport. The combined enhancement of these parameters is expect to lead to elongated spin diffusion lengths in CMHS with larger layer numbers.

To leverage the enhancements in spin lifetimes and carrier mobilities, we made circularly polarized light (CPL) detectors based on the (R/S-MPEA)_2_MA_n‑1_Pb_n_I_3n+1_ series. In CPL detectors, illumination with circularly polarized light (CPL) with angular momentum of ± 1 leads to the generation of spin-polarized carriers with one spin orientation (optical spin injection). Charge transport in the CMHS depends sensitively on both the carrier spin sense and the chirality of the CMHS via the chiral-induced spin selectivity (CISS) mechanism, whereby the material selectively transmits carriers of a specific spin orientation, determined by the enantiomer of the chiral spacer.^16,30^ This mechanism is expected to lead to anisotropic transport of spin-polarized carriers, giving rise to polarization-dependent photocurrent.

The CPL detectors have a standard inverted photodiode architecture of ITO | MeO-2PACz | CMHS | PC_61_BM | BCP | Al as depicted in Figure 5a, with an ∼250 nm CMHS layer (Figure 5b). The key advantage over the photoconductor architecture is the presence of selective contacts imposed by the transport layers and electrodes with differing work functions, which drives the separation of carriers without applied bias, similar to a perovskite solar cell. In contrast, lateral photoconductor structures often require large bias values to enable CPL detection, which may lead to dramatic changes in band structure or carrier properties as a function of voltage, making it difficult to pinpoint the contribution of intrinsic spin transport capability. The fabricated active layers have a similar phase-composition to those used in the spectroscopy study (Figure S14a), and a horizontal orientation (Figure S14b). The red-shifted absorbance and carrier mobility of the *n* = 3 CPL detector resulted in a broad spectral window of 400–700 nm, with a larger EQE (Figure 5c), responsivity and specific detectivity (Figures S15a-c) under unpolarized light, reaching a maximum of 7.83 × 10^12^ Jones between 460 and 604 nm. This specific detectivity is among the highest reported for CMHS-based photodiodes under self-powered conditions. Additionally, the line shape of the EQE clearly shows that there is a contribution to the response from the impurity phases (i.e, different *n*’s than that of the target values) in the *n* = 2 and *n* = 3 photodetectors (at their respective excitonic resonances around c.a. 609 and 645 nm) but they have a low contribution toward the total photocurrent generated, especially compared to the dominant phase, and should not contribute significantly to polarization anisotropy in photocurrents (*vide infra*).

**Figure 5:**
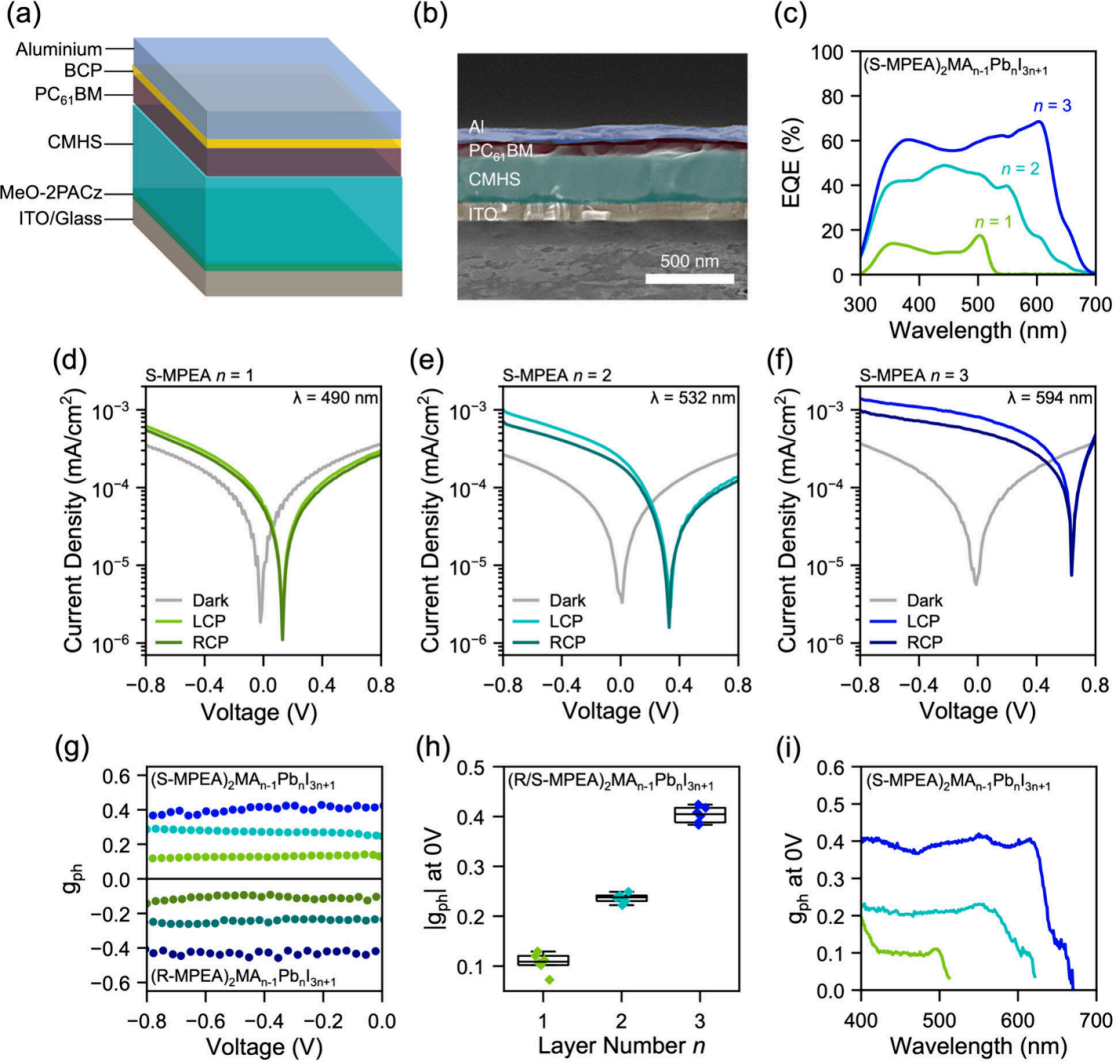
Schematic representation of CPL detectors with a photodiode-type architecture (a). Cross-section SEM image of a representative CPL detector stack with false color to denote the layers used (b). External quantum efficiency (EQE) of CPL detector based on (S-MPEA)_2_MA_n‑1_Pb_n_I_3n+1_ measured under short-circuit conditions (c). J–V traces under dark conditions, and left/right circularly polarized light of CPL detectors with a layer number of 1 (d), 2 (e) and 3 (f). Anisotropy of photocurrent g_ph_ over a bias range of −0.8 to 0 V (g) and statistical distribution of g_ph_ values under self-powered conditions (h) of (R/S-MPEA)_2_MA_n‑1_Pb_n_I_3n+1_ based photodiodes. The green, cyan and blue traces correspond to a layer number of 1, 2 and 3 respectively, while the lighter and darker hue denote S- and R- enantiomers respectively. (i) Spectral distribution of g_ph_ of (S-MPEA)_2_MA_n‑1_Pb_n_I_3n+1_ photodetectors under short-circuit conditions measured via EQE under CPL illumination.

The J–V characteristics of the S-MPEA based CPL photodetectors with varying *n* are depicted in Figure 5d-f. All photodiodes show consistently low dark current densities, around 3 × 10^–4^ mA/cm^2^ at −0.8 V. We performed polarization-dependent J–V measurements by exciting the *n* = 1, 2, and 3 photodiodes at 490 nm, 532 and 594 nm, slightly above their excitonic peaks, and near the crossover point in the CD spectra. The photoexcitation response was found to vary depending on the polarization of the light source and chiral enantiomer. We calculated the anisotropy factor of photocurrents, g_ph_, eq 1, where J_LCP_ and J_RCP_ are the photocurrent density generated upon illumination with LCP or RCP light of a specific wavelength:

1 $$g_{ph}=\frac{2\cdot \left(J_{LCP}-J_{RCP}\right)}{\left(J_{LCP}+J_{RCP}\right)}$$

Thus, the maximum *g*_*ph*_ can reach ±2. The photodiode architecture enabled self-powering, with noticeable differences in photocurrents under 0 V. Curiously, the anisotropy of photocurrents g_ph_ increased as a function of layer number, from ± 0.13 for *n* = 1, to ± 0.25 for *n* = 2, reaching a maximum of ± 0.43 for *n* = 3, showing an analogous trend to the spin lifetime and OPTP measurements. We found that the g_ph_ did not vary significantly as a function of reverse bias voltage, showing only a slight increase at −0.8 V (Figure 5g). When the opposite enantiomer, R-MPEA, was employed, the photoresponse exhibited an identical trend in g_ph_ (Figure S16a-c) under zero-bias and reverse-bias, but with the R-enantiomers delivering larger photocurrents under RCP as compared to LCP, i.e, the opposite behavior. On the other hand, no polarization distinguishability is observed in the vicinity of and beyond the open-circuit voltage, irrespective of composition, as under forward bias conditions there is severe recombination of photogenerated carriers with the injected current. As a result, the injected current dominates the response, with the contribution of photogenerated carriers being reduced to zero, resulting in a weak polarization-dependent photocurrent. However, it is worth mentioning that even though the photocurrent contribution vanishes under forward bias (>*V*_oc_), the dominant injected current will still be spin polarized due to the chiral-induced spin selectivity (CISS) effect as dictated by the chirality of the CMHS. In such a regime, distinguishing between spin-up and spin-down carriers would require the use of a ferromagnetic electrode and a magnetic field to allow extraction of carriers with a specific spin orientation similar to a CISS spin valve^30^ To validate the systematic increase in CPL distinguishability as a function of layer number, we checked the statistical reproducibility of our CPL detectors by evaluating the g_ph_ at 0 V for six independent devices per composition. The average g_ph_ was determined to be 0.11 ± 0.02, 0.24 ± 0.01, and 0.40 ± 0.02 for the *n* = 1, 2, and 3 photodectors respectively (Figure 5h).

In addition, we examined the spectal dependence of g_ph_ in the S-MPEA-based photodetectors via EQE measurements under circularly polarized light illumination (CP-EQE, Figure 5i). The light source is monochromated light from a xenon lamp, and was subsequently circularly polarized using a linear polarizer and quarter waveplate (Figure S17). The EQE values were calibrated with respect to a reference silicon photodiode illuminated using the above configuration, with a negligible difference in measured voltages when the quarter waveplate is set to either +45 or −45 (Figure S17b). This calibration confirms that right- and left-handed light fluences reaching the photodetectors are essentially equivalent, and thus any differences in the measured EQE arise purely from the polarization-dependent photoresponse and are dictated by the spin sense aligned with the different enantiomers. Consistent with the J–V measurements, the S-MPEA photodetectors exhibit noticeably larger EQE values upon illumination with LCP compared to RCP (Figure S18a-f). The g_ph_ values at 490 nm, 532 and 594 nm were similar to those derived via J–V measurements in Figure 5d-f. The g_ph_ of our CPL detectors is nearly spectrally invariant leading up to the excitonic band edge of the dominant *n*, whereafter it drops slightly. The *n* = 3 CPL detector in particular possessed a considerable g_ph_ of ∼0.4 up to 610 nm, broadening the spectral window up to which CPL detection is possible. This is surprising, as in contrast to spectral invariance in g_ph_, the g_CD_ spectrum (differential absorption of LCP vs RCP) of the *n* = 3 film is nearly zero between 500 and 570 nm and reaches a maximum between 590 and 610 nm. Thus, the photocurrent response is not solely governed by the differential absorption of CPL, which will be elaborated upon in the following section. Lastly, control experiments on *n* = 3 (R-MPEA)_2_MA_2_Pb_3_I_10_ and (*ra*c-MPEA)_2_MA_2_Pb_3_I_10_ confirmed that the photocurrent response is indeed linked to the chirality of the CMHS, with S-MPEA delivering larger EQE under LCP, R-MPEA under RCP and *rac*-MPEA showing similar EQE spectra irrespective of the illumination polarization (Figure S19a-c).

Interestingly, our CPL detectors show no clear correlation between the magnitudes of the g_CD_ and g_ph_, with the *n* = 3 system showing a noticeably larger g_ph_ but a comparatively lower g_CD_, especially with respect to the *n* = 2 phase. These findings beg the question as to whether a large g_CD_, while certainly beneficial, is a requisite for efficient photocurrent discrimination. Conventionally, it is expected that CMHS with large g_CD_ factors display strong differential absorption of CPL of a specific polarization, giving rise to unbalanced population in of carriers for the different polarizations and subsequently a large g_ph_.^14,48^ It is worth noting that the g_CD_ values, however, are on the order of 10^–3^ to 10^–1^ (Table S2) in terms of differences in optical density for LCP vs RCP measured by typical spectrophotometers, signifying a very small difference in the absorption of left- and right-handed light. However, there is a huge disparity between the reported g_CD_ values in CMHS (10^–4^ to 10^–2^) compared to the reported anisotropy of photocurrents g_ph_ which lie between ± 0.1 to ±1.1.^14,49–51^ Thus, other processes in the light driven polarized photocarrier generation, transport and collection must be at play in addition to slight absorption anisotropy of LCP vs RCP depending on the enantiomer. In fact, from our results here, the CD plays a very minor to no role in the discrimination between left-handed and right-handed light.

We posit that the spin lifetime and/or spin-dependent transport plays the major role in detecting the difference in light polarization. Circularly polarized light will produce spin-polarized carriers depending on the handedness of the light and not determined by the chirality of the absorber. The optically generated spin-polarized carriers must then have different lifetimes and/or transport dynamics that depend on chirality in order to discriminate between carriers with different angular momentum. Based on a drift-diffusion and absorption model, the ratio of spin-polarized photocurrents can be represented as a function of performance parameters such as differential absorption, spin-polarized carrier mobility and the spin lifetime of carriers with different spin orientation (controlled by the polarization of CPL). The relationship at zero-bias is denoted by eq 2 (a detailed derivation and assumptions are presented in Supplementary Note 2).

2 $$\frac{I_{LCP}}{I_{RCP}}=\left[\frac{\left(1-10^{-A\cdot \left(1+\frac{g_{CD}}{2}\right)}\right)}{\left(1-10^{-A\cdot \left(1-\frac{g_{CD}}{2}\right)}\right)}\right].\left[\frac{\mu_{LCP}.\tau_{LCP}^{s}}{\mu_{RCP}.\tau_{RCP}^{s}}.\frac{\left[1-e^{-L/\mu_{LCP}.\tau_{LCP}^{s}.E}\right]}{\left[1-e^{-L/\mu_{RCP}.\tau_{RCP}^{s}.E}\right]}\right]$$

I_LCP_ and I_RCP_ refer to spin-polarized photocurrents generated under left- and right-circularly polarized light, respectively. A^LCP^ and A^RCP^ denote the optical density of the chiral perovskite under LCP and RCP. The optical density can be correlated to the g_CD_ as explained in supplementary note 2. μ_LCP_ and μ_RCP_ are the mobilities of spin-polarized carriers generated under LCP and RCP post angular-momentum transfer Δ*m*_*J*_ = ±1, while τ^s^_LCP_ and τ^s^_RCP_ are the respective spin lifetimes. L is the photoactive layer thickness, and E is the electric field in V/cm.

For a fixed enantiomer (e.g., (S-MPEA)_2_PbI_4_), g_ph_ shows a weak scaling with g_CD_, (1st term in brackets in eq 2) with the g_ph_ becoming comparable to those in literature only at g_CD_ > 1 (Figure 6a). These modeling results are in line with previous reports on silver metamaterials with modest spin–orbit coupling, which enjoy an extraordinary g_CD_ of 0.9 and a g_ph_ of 1.09.^52^ However, it is clear that the typical g_CD_ is not sufficient to generate a population imbalance of photocarriers to warrant the large disparity in I_LCP_ and I_RCP_ in one enantiomeric state in the context of structurally chiral metal-halide perovskites.

**Figure 6:**
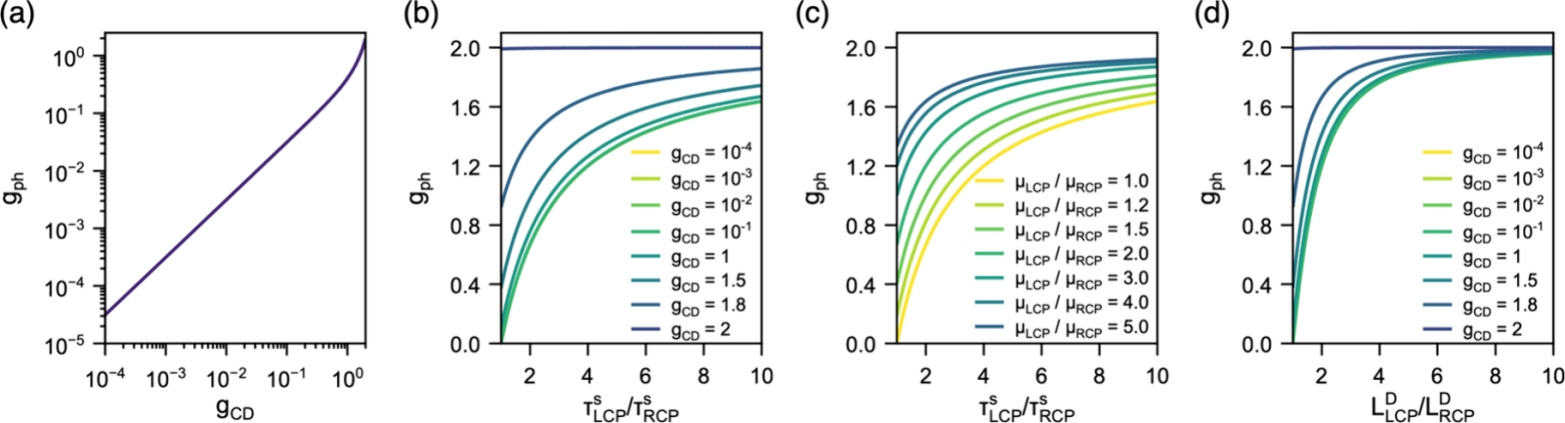
Modeling anisotropy of spin-polarized photocurrent as a function of (a) g_CD_, (b) g_CD_ and ratio of spin-polarized carrier mobility, (c) ratios of spin lifetime and spin-polarized carrier mobility, and (d) g_CD_ and ratio of spin diffusion length under LCP and RCP illumination. Illumination of a chiral enantiomer with LCP (+1) or RCP (−1) leads to the formation of spin-polarized carriers after angular momentum imparted via optical spin injection.

On the other hand, the inclusion of chiral organic spacers in the lattice leads to symmetry breaking and Rashba splitting in the conduction band minimum (CBM).^11^ As a result, the spin-up and spin-down carriers reside in different conduction bands, relax at different rates and possess distinct transport phenomena.^53^ Thus, both spin lifetime and mobility are expected to vary with the spin orientation of the carrier for chiral semiconductors. Our modeling suggests that even a small difference in carrier mobility (Figure 6b) and spin lifetime (Figure 6c) with different spin orientation can lead to appreciable change in g_ph_, due to the preferential collection of spin-up or spin-down carriers. For instance, to achieve a g_ph_ = ±0.4, if the spin-up and spin-down mobilities are the same, a spin lifetime ratio of ∼1.5 is needed; similarly, if the spin-lifetimes are similar, then a ratio of the mobilities would also be ∼1.5. Such discrimination in transport of spin-up and spin-down depending on chirality has already been observed in chiral perovskites and attributed to CISS.^54–56^ It is worth noting that while the relatively low g_CD_s of CMHS do not contribute significantly into determining g_ph_, enhancing g_CD_ beyond 10^–1^ via metastructural design or nanoscale morphologies coupled with Rashba splitting can make larger g_ph_ more accessible.^54,55^ Incorporating these strategies to study the capability of such architectures toward polarization-dependent photocurrent discrimination can be a subject of future research.

Finally, we stress the importance of a key descriptor of spin transport in Figure 6d: the spin diffusion length, L_D_, which describes the distance up to which a spin-polarized carrier may traverse before the spin dephases. Spin diffusion length is denoted by

$$L_{D}=\sqrt{\mu \cdot \frac{k_{B}\cdot T}{q}\cdot \tau^{s}}$$ 3

k_B_ is the Boltzmann constant, *T* the temperature, and q represents the elementary unit of charge. As shown in Figure 6d, it is clear that even a small difference in spin diffusion length under different polarization can drive the carrier transport sufficiently to allow appreciable discrimination between photocurrents. It is possible to leverage differences in spin diffusion lengths between different polarized carriers by modifying the architecture (e.g., adjusting the active layer thickness such that the spin-diffusion length of one spin orientation exceeds the film thickness while that of the opposite spin is shorter, thereby favoring selective carrier collection and maximizing g_ph_). Indeed previous focus on optimization of CPL detectors has revealed an active layer thickness dependence of g_ph_ where active layers with nonoptimal film thicknesses display inferior g_ph_ compared to thicker active layers.^50^ Thus, we emphasize that while generating highly asymmetric carrier populations through selective CPL absorption is certainly important, preserving the spin lifetime during carrier diffusion and driving the spin-polarized carriers to collection is more critical toward high-performing CPL detecting structures.

## Conclusions

We investigated the layer number dependence of chiroptical activity, spin lifetime and carrier mobility in quasi-2D CMHS. Increasing the layer number elicits systematic enhancements in spin lifetime and carrier mobility, promoting longer spin-diffusion lengths. However, this occurs at the cost of chiroptical activity, where we observed lower g_CD_ in *n* = 3 thin films possibly due to reduced octahedral distortions. Nevertheless, prototype *n* = 3 CPL detectors deliver high responsivity with an appreciable anisotropy of photocurrent of 0.44 at 594 nm, outperforming the *n* = 1 and 2 photodetectors, which have greater g_CD_. The improved mobility, especially along the in-plane direction, of quasi-2D CMHS offers a new material platform to reliably interrogate the anisotropy in spin transport, with possibilities to align the screw-axis with the direction of applied field in say, semiconductors in the *P*2_1_ space group. In addition, we note that the spin diffusion length may be crippled due to the limited mobility along the out-of-plane direction. Optimizing the crystal orientation may afford improvements to spin transport, and will be the focus of future work.

## Supplementary Material



## Notes

Experimental methods. Chiral spacer screening for quasi-2D CMHS formation and relevant characterization. SCXRD of the inorganic framework of *n* = 3 (R-MPEA)_2_MA_2_Pb_3_I_10_. Properties of *n* = 1–4 CMHS crystals. Orientation, morphological and chiroptical properties of *n* = 1–3 CMHS thin films. Comparison of TA and TRCD spectra at 1 ps, fluence-dependent TRCD transients, and resonant vs 400 nm excitation OPTP kinetics of *n* = 1–3 CMHS thin films. Additional UV–vis, XRD, EQE, J–V and CP-EQE spectra of *n* = 1–3 (R-/S-/rac-MPEA)_2_MA_2_Pb_3_I_11_ CPL detector devices. Comparison of g_CD_ and spin lifetimes of *n* = 1–3 CMHS with other reported materials. Modeling of CPL detector discrimination factors for vertical photodiode and lateral photoconductor architectures. (PDF)
