# Supplementary material for: Layer Number Dependence of Chirality and Spin Polarized Lifetime in Chiral 2D Halide Perovskites
Source: J Am Chem Soc. 2025 Nov 19;147(48):44457–67. doi: 10.1021/jacs.5c16029 (PMC12679627; doi:10.1021/jacs.5c16029)
Supplement: Supplementary file 1 [file ja5c16029_si_001.pdf]

Supporting Information

Layer number dependence of chirality and spin polarized lifetime in chiral 2D halide perovskites

Shripathi Ramakrishnan,[1,2,†] Yifan Dong,[1,3,†] Yi Xie,[4] Jacob L. Shelton,[1] Matthew P. Hautzinger,[1] Duong Nguyen Minh,[1] Margherita Taddei,[1] Xiaoyu Zhang,[2] Yugang Zhang,[6] David B. Mitzi,[4,5] Md Azimul Haque,[1] Jeffrey L. Blackburn,[1] Qiuming Yu,[2*] Matthew C. Beard,[1*] Joseph M. Luther[1*]

[1]National Renewable Energy Laboratory, Golden, CO, 80401, USA
[2]Robert Frederick Smith School of Chemical and Biomolecular Engineering, Cornell University, Ithaca, NY, 14853, USA
[3]Department of Chemistry, University of California, Riverside, CA, 92521, USA
[4]Thomas Lord Department of Mechanical Engineering and Materials Science, Duke University, Durham, NC, 27708, USA
[5]Department of Chemistry, Duke University, Durham, NC, 27708, USA
[6]Center for Functional Nanomaterials, Brookhaven National Laboratory, Upton, NY 11973-5000, USA

[†]These authors contributed equally.

qy10@cornell.edu, matt.beard@nrel.gov, joey.luther@nrel.gov

Materials

All materials were purchased from commercial suppliers and used without further modification. Methylammonium iodide (MAI, >99.99%) was purchased from GreatCellSolar. Acetonitrile (ACN, anhydrous, 99.9%), tetrahydrofuran (THF, anhydrous, 99.9%), chlorobenzene (CB, anhydrous, 99.9%), toluene (TOL, anhydrous, 99.9%), hydroiodic acid (HI, 57 wt% in H_2O, distilled, stabilizer-free), H_3PO_2 (50 wt% in water), polymethylmethyacrylate (PMMA) and bathocuproine (BCP, 99.99% sublimed grade) were purchased from Sigma Aldrich. Lead iodide (PbI_2, 99.99%, perovskite grade), (*R*)-(+)-β-methylphenethylamine and (*S*)-(-)-β-methylphenethylamine were purchased from Tokyo Chemical Industry TCI America. PCBM was purchased from Nano-C (Quebec, Canada). Aluminum (Al, 99.999%) was purchased from R.D. Matthis.

CMHS single-crystal growth and characterization

Single-crystals of $(R\text{-MPEA})_2MA_{n-1}Pb_nI_{3n+1}$ perovskites were synthesized by a typical slow cooling method. R-MPEA, MAI and PbI_2 were dissolved in aqueous HI solution as per the concentrations in Table S1 for n = 1 – 4 CMHS. The phase-purity and distribution of arising crystals was found to be very sensitive to the concentrations of precursors. S-MPEA crystals were grown using an identical process, but by replacing R-MPEA with S-MPEA. The solutions were heated to 100°C in an aluminum heatingblock and subsequently cooled to room temperature at a rate of 1°C/h to achieve phase-purity. The crystals are thoroughly dried via vacuum filtration for at least 1 h. To expel residual solvents from the lattice, the crystals are annealed at 100°C for 10 mins and subsequently dried in a vacuum oven at 60°C overnight.

Film preparation

CMHS single-crystals were dispersed in a 8:2 v/v mixture of ACN and THF at an initial concentration of 100 mg/mL. 2 wt% MACl (with respect to mass of single-crystals) was added to the solvent mixture. This mixture was mixed using a shaker for 10 min to form a pale-yellow solution. The solution was used without filtration. For thinner films, the precursor can be diluted with ACN prior to spin-coating. 10-20 µL aforementioned solutions were dynamically spin-coated on a glass substrate at 4000 rpm for 20 s, with the perovskite phase crystallizing upon impact. The film was then annealed at 100°C for 15 min to produce films with nearly the same composition as the single-crystals. Before characterization, 20 mg/mL of PMMA in toluene was deposited atop the 2D-MHPs at 4000 rpm for 10 s.

Photodiode-type Circularly Polarized Light (CPL) detector fabrication

ITO substrates were sequentially sonicated in tap water with 1 vol% Hellmanex solution, DI water, acetone and isopropanol for 10 min each respectively, followed by UV-ozone treatment for 10 min. Subsequently, the ITO substrates were transferred into an N_2-filled glovebox. 100 µL of MeO-2PACz (1 mmol/mL in ethanol) was allowed to stand on the substrate for 5 seconds, followed by spin-coating at 4000 rpm for 30 s and annealing at 100°C for 10 min. 2D-MHPs were deposited using the methodology listed in the film preparation section to form films with an abundance of target n. Then, $PC_{61}BM$ (20 mg/mL in CB) is spin-cast onto the stack at 1000 rpm for 30 s. Finally, the samples were loaded in a thermal evaporation chamber for BCP (2 nm at 0.2 Å/s) and aluminum (100 nm at 1 Å/s)

deposition at a pressure of 2 x 10^{-6} torr. The final device area is 0.09 cm^2 as defined by the overlap between the evaporation mask and aluminum electrode.

CPL Detector Characterization

A combination of a linear polarizer and quarter waveplate was used to convert linearly polarized laser light (continuous wave) into circularly polarized light for the I-V measurements. The power density was maintained at 8.1 x 10^{18} cm^{-3}, 7.0 x 10^{-17} cm^{-3} and 7.7 x 10^{-17} cm^{-3} for 494, 532 and 594 nm light sources respectively. The I-V characteristics were measured using a Keithley 2425 sourcemeter and controlled using a Matlab code. The external quantum efficiency (EQE) measurement was performed using an Oriel 200 QE system with a 150 W Xenon-lamp as the light source and measured under short-circuit conditions.

CP-EQE Measurement

The CP-EQE experiment was performed at Cornell University. A 150 W Xenon-lamp is the light source. The calibration is performed using a power meter to read out the power density of different wavelengths of monochromated light. First, a pair of linear polarizers (90° apart) was placed between the light source and power meter to transmit only CPL. A quarter waveplate was introduced between the two linear polarizers. The fast-axis was found to be ±45°. To calculate the degree of circular polarization, the second linear polarizer was swept between 0 to 180° to obtain the maximum and minimum power, which was then used to determine the degree of circular polarization. We observed that the degree of circular polarization was quite consistent across the 400-850 nm region. For the actual device measurements, the second linear polarizer was removed to increase the light intensity.

Optical and XRD Characterization

XRD measurements were performed using Rigaku Ultima IV with Cu Kα radiation at ambient temperature. Scanning electron microscopy images were taken on a Hitachi S-4800 scanning electron microscope. Ultraviolet (UV)–visible absorption spectra were taken on a Cary 7000 spectrometer. Photoluminescence spectra (PL) were collected using a Horiba PL spectrometer at an excitation wavelength of 405 nm. CD measurements were carried out using an Olis DSM 170 spectropolarimeter.

Single-crystal X-ray Diffraction

Data for the n = 3 $(R\text{-}MPEA)_2MA_2Pb_3I_{10}$ system was collected on a Rigaku XtaLAB Synergy-S diffractometer (Mo Kα, λ = 0.71073 Å; 50 kV, 30 mA) at 115 K. To mitigate stacking disorder, crystals were gently cleaved with adhesive tape, and residual thin fragments were removed with diethyl ether, yielding thinner crystal layer for SC-XRD. Peak hunting, data reduction, and numerical absorption correction for collected data were performed using CrysAlisPro. The crystal structure was solved and refined using SHELXS direct methods and SHELXL least-squares method within Olex2. Electron-density maps showed severe cation disorder, consequently, only the inorganic n = 3 slab could be modeled reliably, and the organic cations were not refined. Equatorial I sites were treated as split positions (occupancies ~0.55 and 0.45).

Grazing-Incidence Wide-Angle X-ray Scattering

The grazing incidence wide-angle X-ray scattering (GIWAXS) measurements were conducted at the Soft Matter Interfaces beamline (12-ID) of the National Synchrotron Light Source II (NSLS-II) at Brookhaven National Laboratory. The X-ray beam, with an energy of 16.1 keV ($\Delta E/E$ = 0.01%), was focused to a dimension of 200 um (horizontal) x 30 um (vertical). Scattered X-rays were collected using a PILATUS3 900 kW detector (Dectris, Switzerland) with a 1475 × 619 array of 0.172 mm square pixels. Thin film samples (about 1 cm x 1 cm) were mounted on a piezo-motorized stage. For each sample, measurements were taken at three different positions with an exposure time of 1 second. The 2D GIWAXS maps displayed in Figure 2 have a grazing-incidence angle of 0.5 degrees. To obtain a wide range of wave vector transfer (q), a series of 2D diffraction patterns were collected by rotating the detector along an arc at a sample-to-detector distance of 275 mm. The resulting patterns were stitched together using in-house software. Silver behenate was used as a standard for scattering angle calibration. The acquired data was reduced using a Python script developed by the NSLS-II staff members. The individual circularly averaged frames, baseline correction and maps of scattering patterns for a specific sample were visualized using a custom Python script developed by the lead author.

Time-resolved Circular Dichroism (TRCD) Spectroscopy

A Ti:Sapphire regenerative amplifier (Coherent Astrella) was used to generate a 1-kHz train of 800-nm pulses (100 fs). A beamsplitter was used to direct approximately 10 µJ toward the probe generation stage consisting of a motorized delay stage, while the remaining portion was used to pump an optical parametric amplifier (OPA, Quantronix PalitraDuo) to generate tunable pump pulses. For the probe, the fundamental 800-nm pulse was focused onto a sapphire plate to generate a visible supercontinuum (420-750 nm). The pump pulse was modulated by an optical chopper (500 Hz) before being focused onto the sample at a 2° angle of incidence relative to the probe with a CaF_2 lens. A broadband linear polarizer and a broadband quarter waveplate were used to generate circularly polarized pump pulses. The now elliptically polarized transmitted probe pulses were collected and collimated by a second CaF_2 lens and were directed toward a quarter waveplate mounted on a motorized rotation stage, which modulates the orientation of the fast axis between +/- 45 degrees relative to the initial probe polarization (100 Hz). As such, the quarter waveplate is always aligned such that it linearly polarizes the elliptical component of the probe pulses, while the achiral background became circularly polarized. A Wollaston prism then separated the beam into two orthogonally polarized arms that were directed to two fiber-coupled broadband detectors. Balanced detection gave rise to a differential signal that reflects only the TRCD response, absent of the TA background. In addition, the TA spectra were also obtained by summing the intensity response of both detectors. The n = 1 films were excited with a pump excitation energy of 485 nm (2.55 eV), n = 2 with 520 nm (2.38 eV) and n = 3 with 590 nm (2.1 eV).

Optical Pump THz Probe (OPTP) Spectroscopy

The fundamental laser pulse (800 nm) was generated by a Ti:sapphire amplifier (Coherent Astrella, 1 kHz repetition rate, 100 fs pulse duration). This pulse was split into two parts where one portion was directed to an optical parametric amplifier (Light Conversion, TOPAS) to produce visible pump beams.

The pump was modulated at 500 Hz with a chopper and attenuated using neutral-density filter wheels. The probe beam was divided into two arms. In the first arm, freely propagating THz pulses were generated via optical rectification using optical rectification in a 1-mm thick <110> ZnTe crystal, focused onto the sample, transmitted, and then refocused onto a detector crystal. These THz pulses were spatially and temporally overlapped with the optical pump at the sample position, with the relative delay controlled by a mechanical delay stage. In the second arm, a gating pulse was employed for free-space electro-optic sampling of the THz pulses with a 0.5-mm thick <110> ZnTe crystal. The gating pulse and the transmitted THz pulse were overlapped both spatially and temporally at the detector crystal to enable detection.

Table S1. Summary of precursor quantities to grow single-crystals of CMHS with layer thickness varying from 1 to 4.

n	R-MPEA (mmol)	MAI (mmol)	PbI_2 (mmol)	HI (mL)	H_3PO_2 (mL)
1	1	0	0.5	3	0.15
2	0.5	1	2	3	0.15
3	0.28	2	3	5	0.25
4	0.1	2.25	3	6	0.3

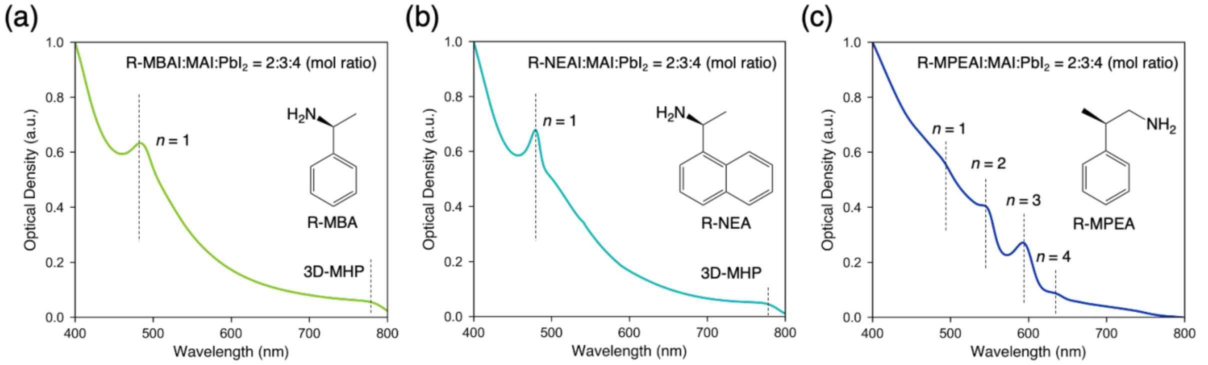

Figure S1. UV-VIS spectroscopy to screen the viability of (a) R-MBAI, (b) R-NEAI and (c) R-MPEAI towards forming *n* > 1 2D-MHPs when spin-casted from a precursor solution with a stoichiometric *n* of <4>.

Supplementary Note 1. Influence of chiral spacer on quasi-2D MHP formation tendency

The tendency to form larger *n* 2D-MHPs is affected by the extent to which the spacers distort the inorganic framework in the initial *n* = 1 structure. Multilayer 2D-MHPs are formed via sequential transformation of smaller *n* into larger *n* through the intercalation of $MAPbI_3$ lattices into the smaller *n* structures during both single-crystal synthesis and film formation.[1,2] Therefore, smaller *n* (*n* = 1) 2D-MHPs with very high Pb-I-Pb bond angle distortion cannot be intercalated with $MAPbI_3$.[3] Selecting an appropriate spacer is a crucial consideration towards layer thickness control of 2D-MHPs. Given the relatively small library of commercially available chiral spacers that are resolved crystallographically, we considered R-1-methylbenzylammonium (R-MBA), R-1-(1-naphthyl)ethylammonium (R-NEA) and R-β-methylphenylethylammonium (R-MPEA) as potential spacers. Before attempting to synthesize phase-pure single-crystals, we sought to assess whether the aforementioned spacers were capable of forming *n* > 1 CMHS. Thus, we prepared thin films from 0.6 M precursor solutions in DMF with a nominal <*n*> of 4 by mixing chiral spacers, methylammonium iodide (MAI) and lead (II) iodide (PbI_2) in a 2:3:4 molar ratio. Precursors with a nominal <*n*> of 4 or 5 prepared from iodide salts have been shown to form larger *n* quasi-2D MHPs due the presence of a larger amount of small A-cation (in this case, MA^+), albeit with a broad phase distribution.[4] UV-VIS absorbance spectra of <*n*> = 4 films with R-MBA, R-NEA and R-MPEA are shown in (**Figure S1a-c**). It is apparent from **Figure S1a-b** that using R-MBA and R-NEA as spacers resulted in a polydisperse mixture of chiral *n* = 1 2D-phase (excitonic peaks at 494 nm and 480 nm respectively) and $MAPbI_3$. No peaks corresponding to *n* > 1 2D-MHPs were observed for these two samples. However, the UV-VIS spectrum of R-MPEA shows prominent peaks corresponding to *n* = 2, 3 and 4, possibly by virtue of the R-MPEA spacer's structural similarity to its achiral counterpart, phenylethylammonium (PEA), a widely studied system. Attempts to grow phase-pure *n* = 3 CMHS single-crystals based on the optimized methods in **Table S1** using R-MBA and R-NEA as spacers had a similar outcome. The resulting precipitates were found to be a mixture of the respective *n* = 1 phase and $MAPbI_3$, as shown in **Figure S2a-b**, corroborating the results from the film formation study. Hence, we investigated R-MPEA further as a model system to design single-crystals and films with high *n* phase-selectivity.

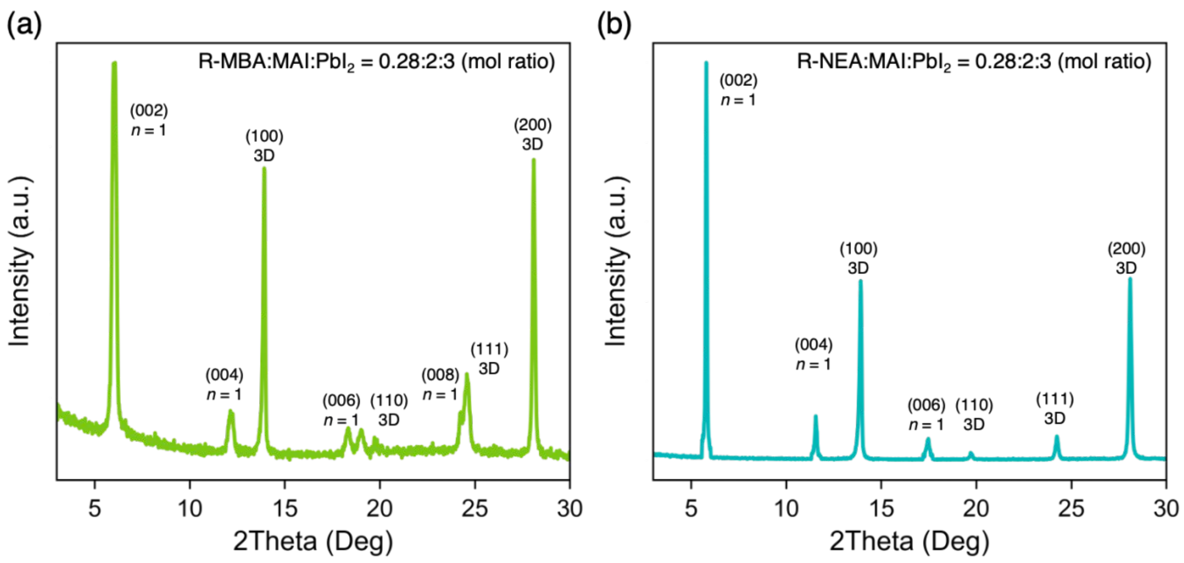

Figure S2. PXRD pattern of single-crystals synthesized using the optimal conditions used to form *n* = 3 CMHS crystals summarized in **Table S1**, when the chiral spacer is (a) R-MBA and (b) R-NEA. It is apparent the crystals thus synthesized yield a mixture of *n* = 1 and 3D-MHP flakes.

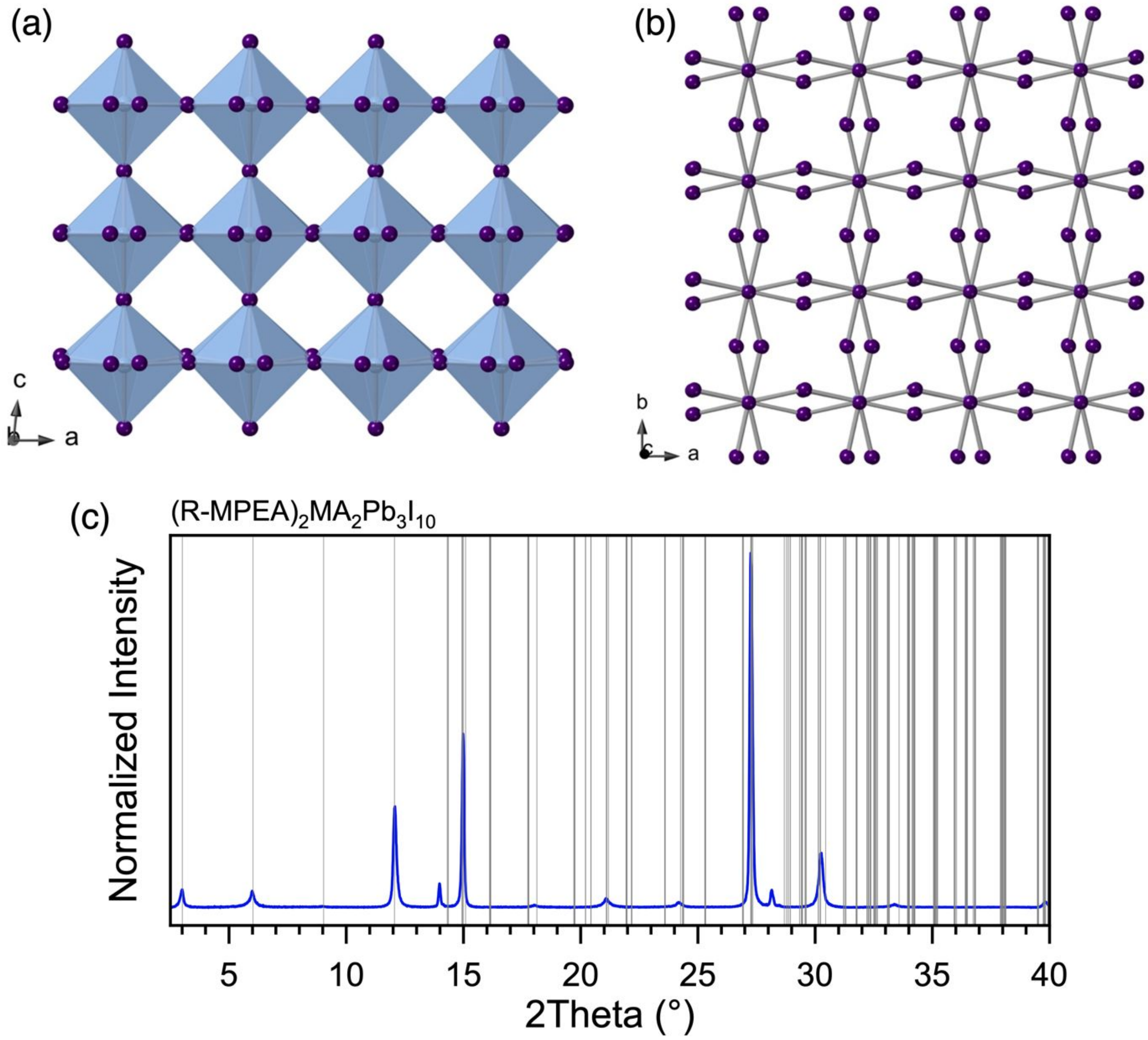

Figure S3. (a) Schematic single-crystal structure of the *n* = 3 inorganic slab, showing corner-sharing $[PbI_6]^{4-}$ octahedra. Stacking is along c. The organic cations are highly disordered and could not be reliably refined. (b) Top view of the n = 3 layer, highlighting positional disorder at the equatorial I sites. (c) Comparison between simulated XRD pattern of above *n* = 3 structure and experimentally obtained PXRD pattern. The blue trace represents the experimentally obtained data, with black vertical lines denoting the peak positions of simulated patterns.

Supplementary Note S2.

To verify the layer thickness of the n = 3 system, we collected single-crystal XRD data at 115 K. Owing to pronounced stacking and cation disorder, only a preliminary model of the *n* = 3 inorganic slab could be refined (**Figure S3**), whereas the organic cations could not be reliably resolved. The equatorial I sites are disordered over two conformations with refined occupancies of appropriately 0.55 and 0.45 respectively. A simulated powder XRD pattern from this model matches the experimental peak positions reasonably (**Figure S3c**), confirming the n = 3 phase in the films. Because the structure remains incomplete and was used solely to confirm layer thickness and phase, we have not deposited it in the CCDC at this stage.

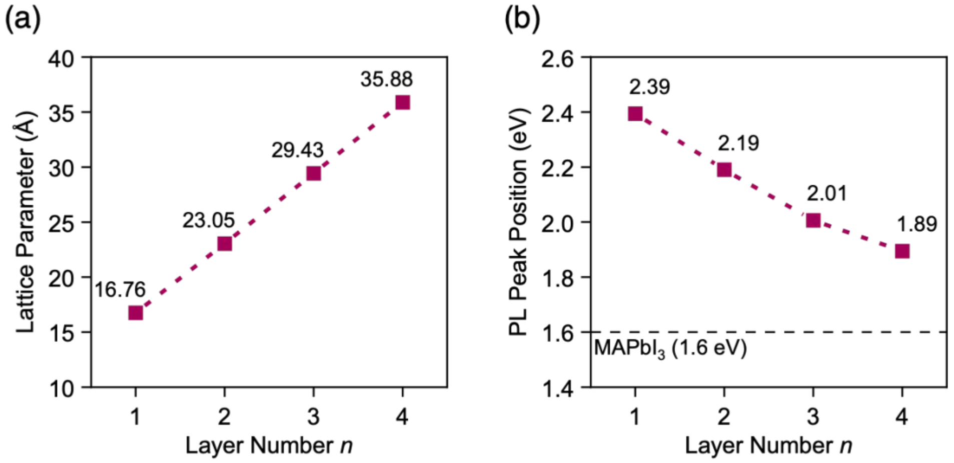

Figure S4. (a) Interlayer $d_{(002)}$ spacing and (b) PL peak position as a function of layer number *n* in (R-MPEA)$_2$MA$_{n-1}$Pb$_n$I$_{3n+1}$.

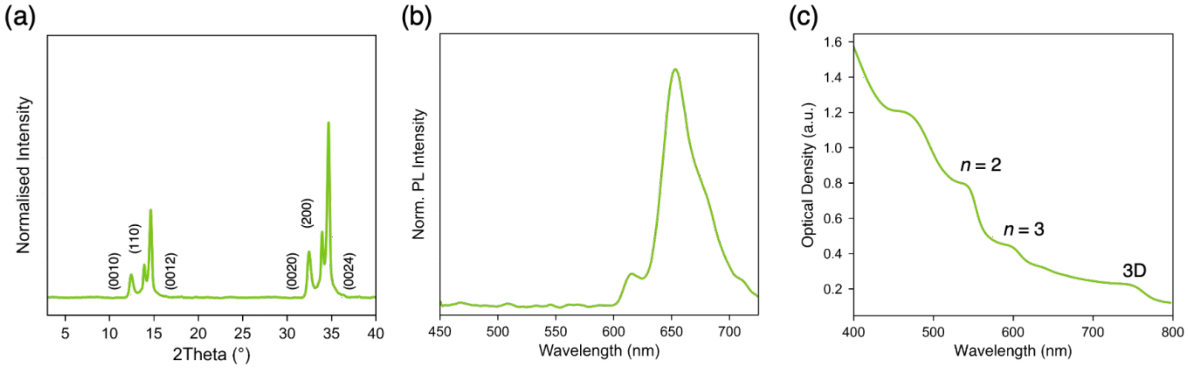

Figure S5. (a) Powder XRD pattern and (b) PL spectrum of attempted n = 4 (R-MPEA)$_2$MA$_3$Pb$_4$I$_{13}$ crystals, and (c) UV-VIS of a thin film prepared by dissolving aforementioned n = 4 crystal in ACN:THF.

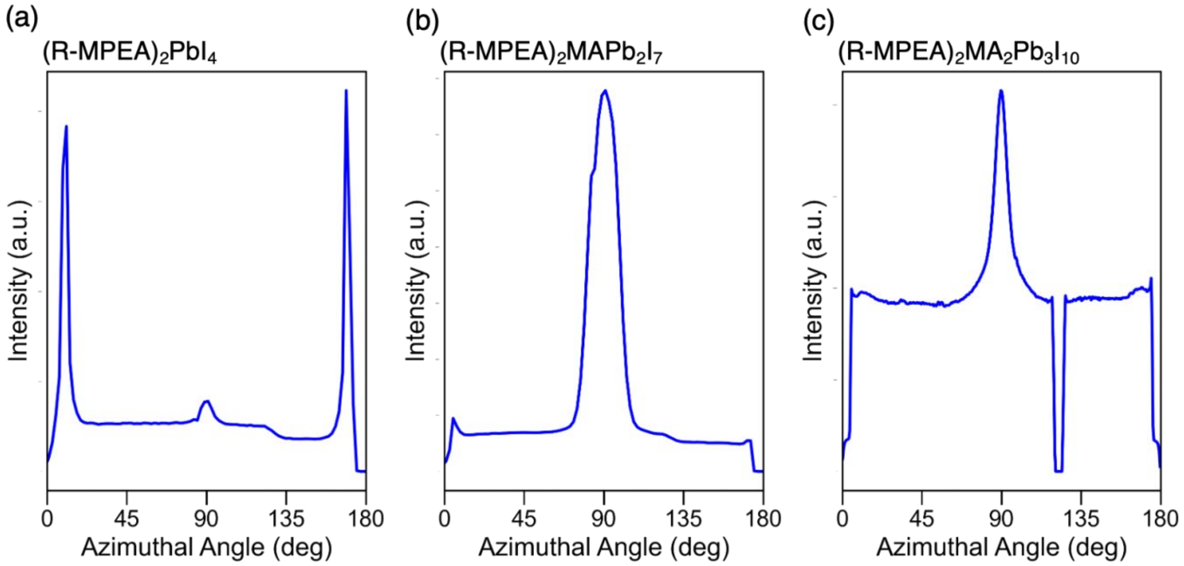

Figure S6. Azimuthal integration of (a) $(R\text{-}MPEA)_2PbI_4$, (b) $(R\text{-}MPEA)_2MAPb_2I_7$ and $(R\text{-}MPEA)_2MA_2Pb_3I_{10}$ thin films along the q = 0.95 – 1.05 $Å^{-1}$ region, depicting the intensity of the (110) peak as a function of angle

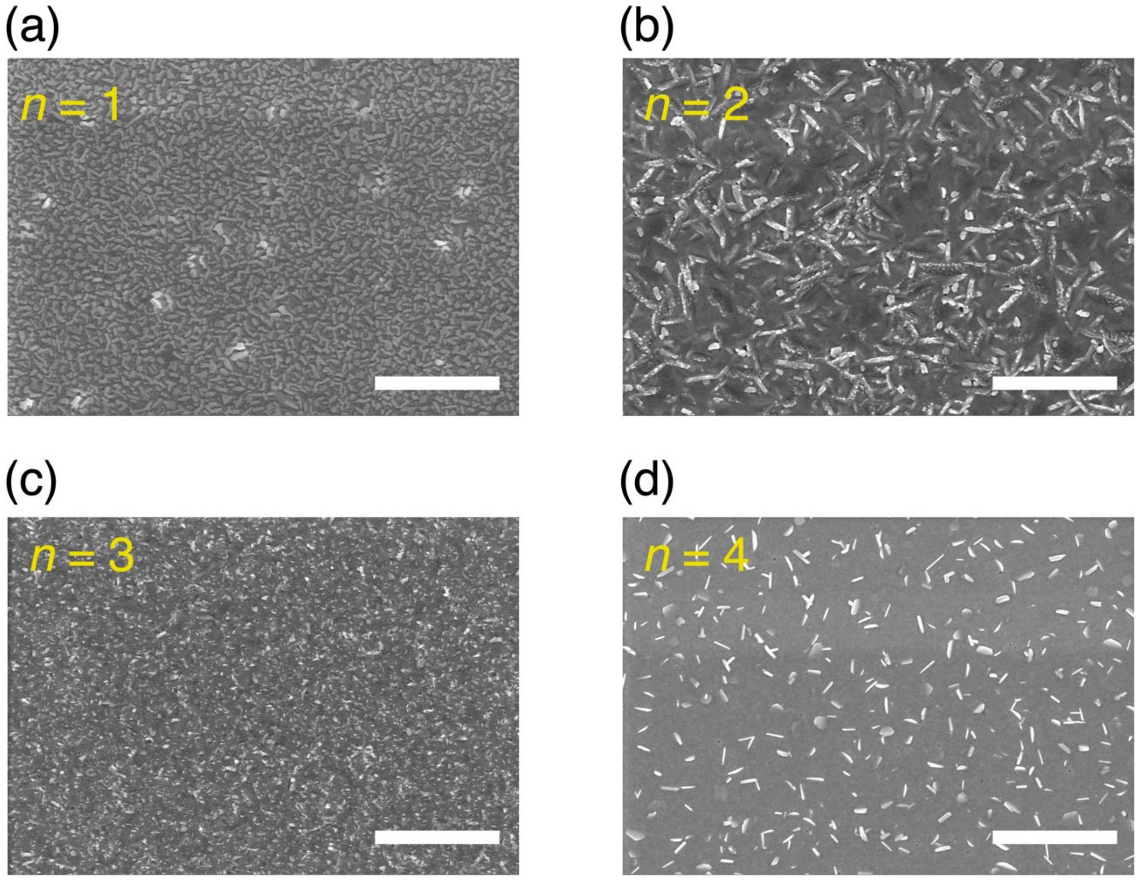

Figure S7. Scanning electron microscopy images of (a) *n* = 1, (b) *n* = 2, (c) *n* = 3 and (d) *n* = 4 (S-MPEA)$_2$MA$_{n-1}$Pb$_n$I$_{3n+1}$ films. The scale bar is 2 μm.

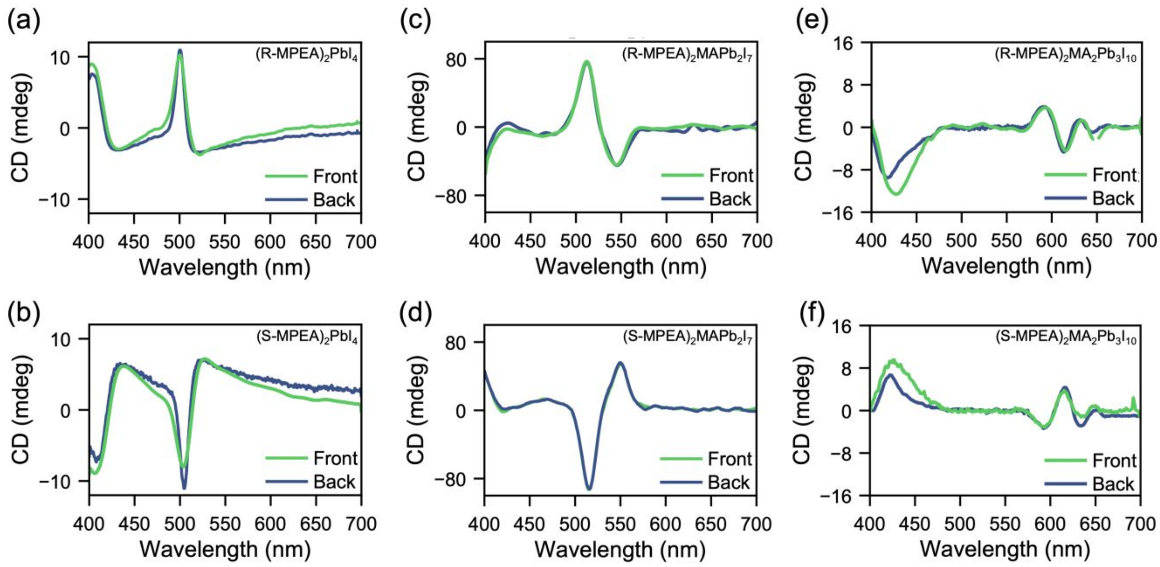

Figure S8. Circular dichroism spectra of comparing spectra captured when illuminated from the front (film side) and back (glass side) for $(R\text{-}MPEA)_2MA_{n-1}Pb_nI_{3n+1}$ (a, c, e) and $(S\text{-}MPEA)_2MA_{n-1}Pb_nI_{3n+1}$ for *n* = 1 (a, b), *n* = 2 (c, d) and *n* = 3 (e, f). All samples show similar CD distribution with respect to different film orientations suggesting minimal interference from asymmetric LDLB interactions.

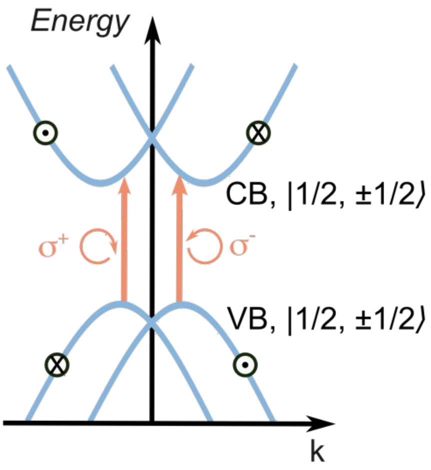

Figure S9. Band diagram depicting the optical transitions in MHPs under circularly polarized excitation.

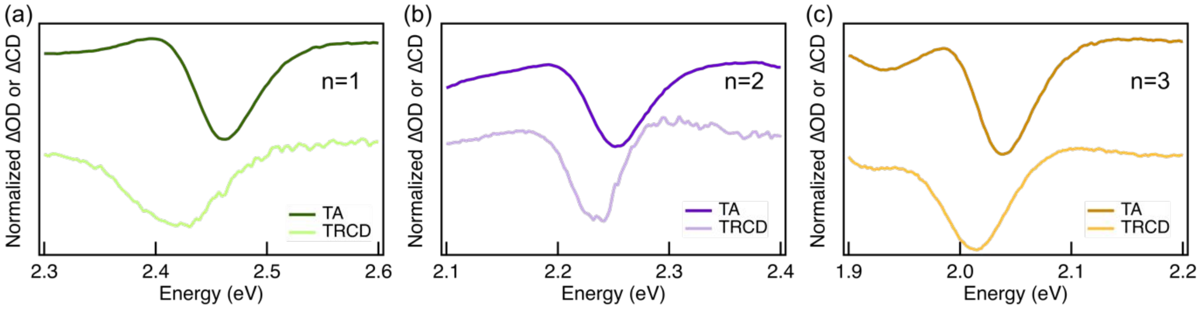

Figure S10. Normalized transient absorption (TA) and time-resolved circular dichroism (TRCD) spectra at 1 ps following photoexcitation for $(R\text{-}MPEA)_2MA_{n-1}Pb_nI_{3n+1}$ films with varying layer thicknesses: (a) n=1, (b) n=2, and (c) n=3.

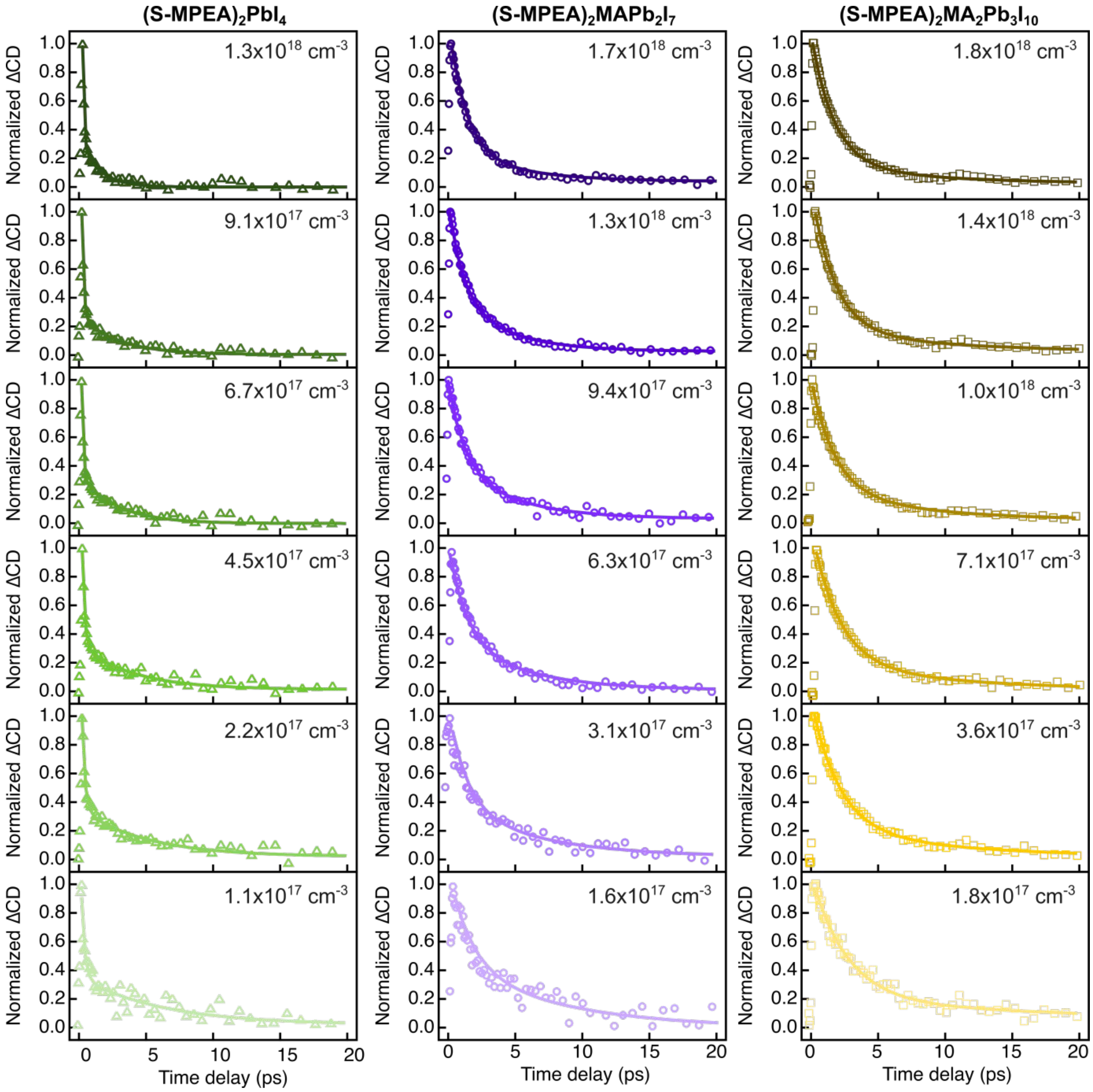

Figure S11. Normalized time-resolved circular dichroism (TRCD) transients for spin lifetimes in thin films with varying layer thicknesses including (a) n = 1, (b) n = 2, and (c) n = 3. The corresponding excitation fluence is included in each panel.

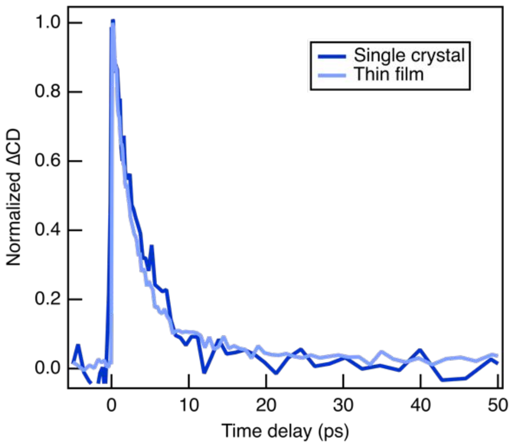

Figure S12. Normalized time-resolved circular dichroism (TRCD) transients for n = 3 showing comparable spin lifetimes between the thin film and single crystal.

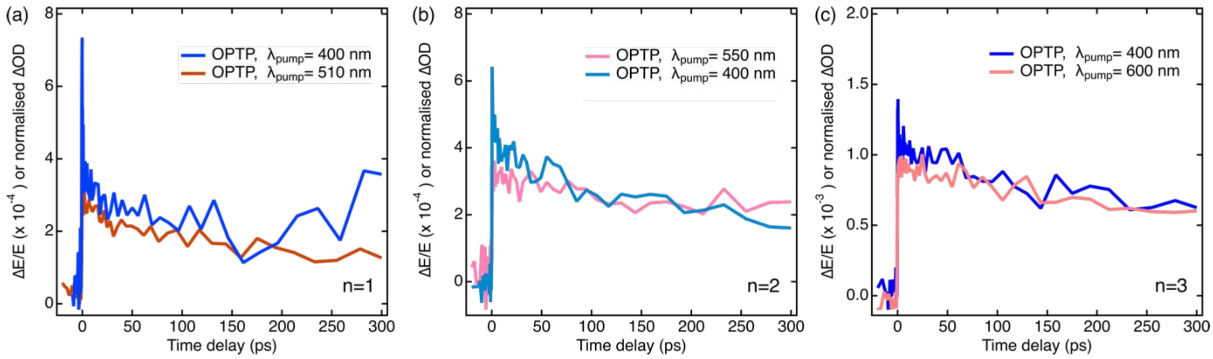

Figure S13. Optical pump terahertz probe kinetics for (a) *n* = 1 at an excitation density of 2.2×10^{17} cm^{-3}, (b) *n* = 2 at an excitation density of 3.1×10^{17} cm^{-3}, and (c) *n* = 3 at an excitation density of 3.6×10^{17} cm^{-3} films excited at 400 nm vs at the band edge.

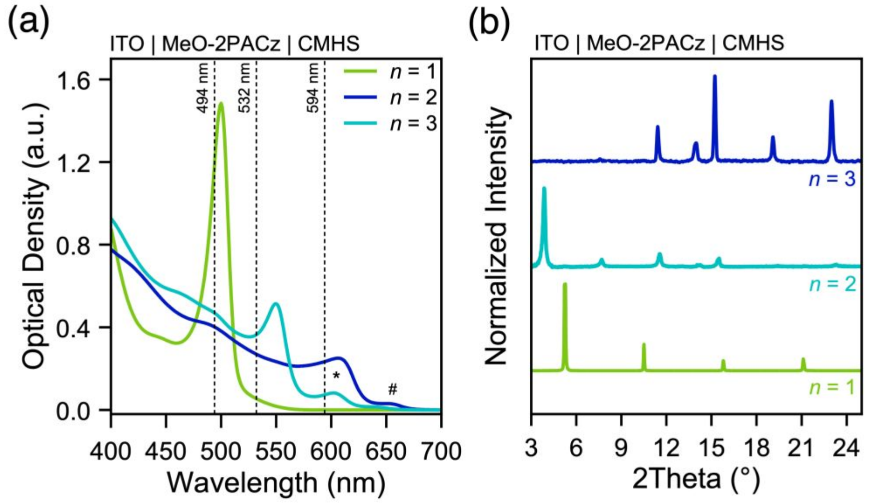

Figure S14. (a) UV-Vis absorption and (b) XRD spectra of $(S\text{-}MPEA)_2MA_{n-1}Pb_nI_{3n+1}$ films deposited on MeO-2PACz, used for CPL-detector fabrication. The black dashed lines on the UV-Vis spectrum denote the wavelengths at which circularly-polarized light detection was performed with the CPL-detector, provided as guidance. Both spectra verify that the major phase present in the films are the intended *n*-value.

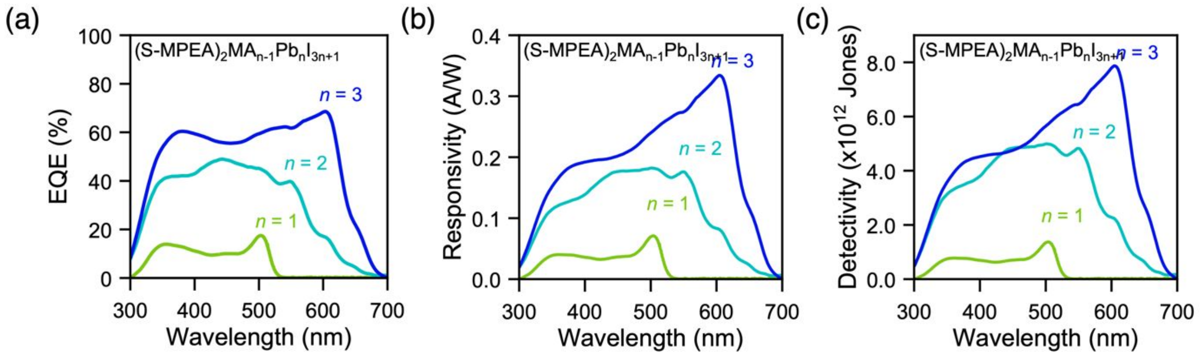

Figure S15. (a) EQE, (b) responsivity, and (c) specific detectivity of CPL-detectors based on (S-MPEA)$_2$MA$_{n-1}$Pb$_n$I$_{3n+1}$.

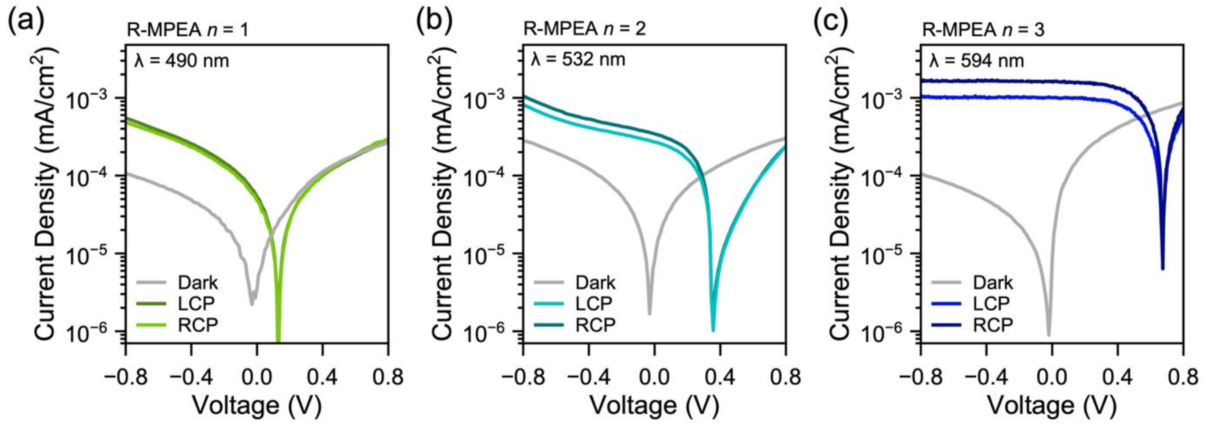

Figure S16. J-V characteristics of $(R\text{-}MPEA)_2MA_{n-1}Pb_nI_{3n+1}$ devices with (a) *n* = 1, (b) *n* = 2, and (c) *n* = 3. Notably, the *R*-enantiomers display the opposite trend as the *S*-enantiomer, producing larger photocurrents under RCP.

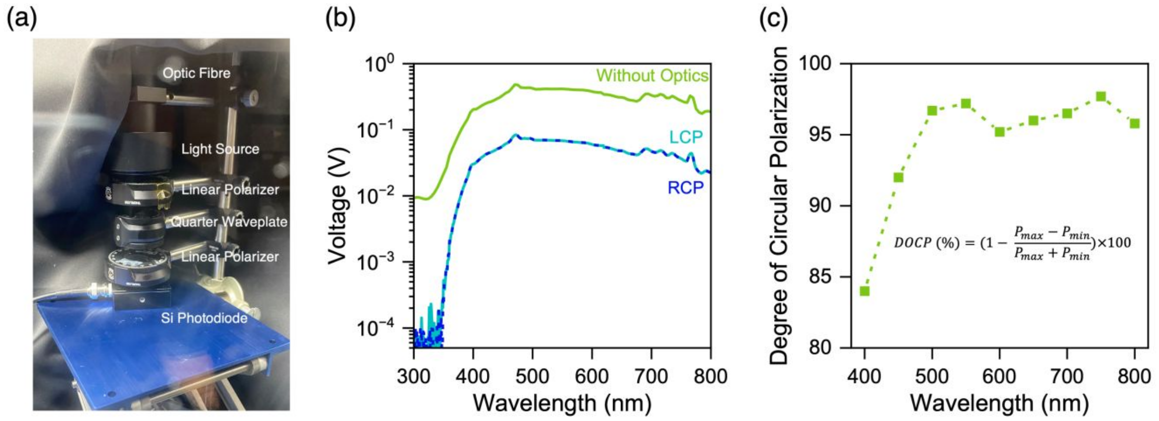

Figure S17. (a) Setup for CP-EQE measurements, with a reference Si photodiode sample mounted. (b) Calibration of reference Si photodiode under regular illumination *sans* optics, under LCP and RCP light. The voltage read out is almost identical for LCP and RCP, signifying that the power densities are very similar under different light polarization, and any differences in EQE will arise purely from the chiral nature of the CMHS device. (c) Degree of circular polarization (DOCP) determined by sweeping the second linear polarizer from 0-180°. The DOCP is generally uniform between 500-800 nm, with a slight dip in the smaller wavelengths.

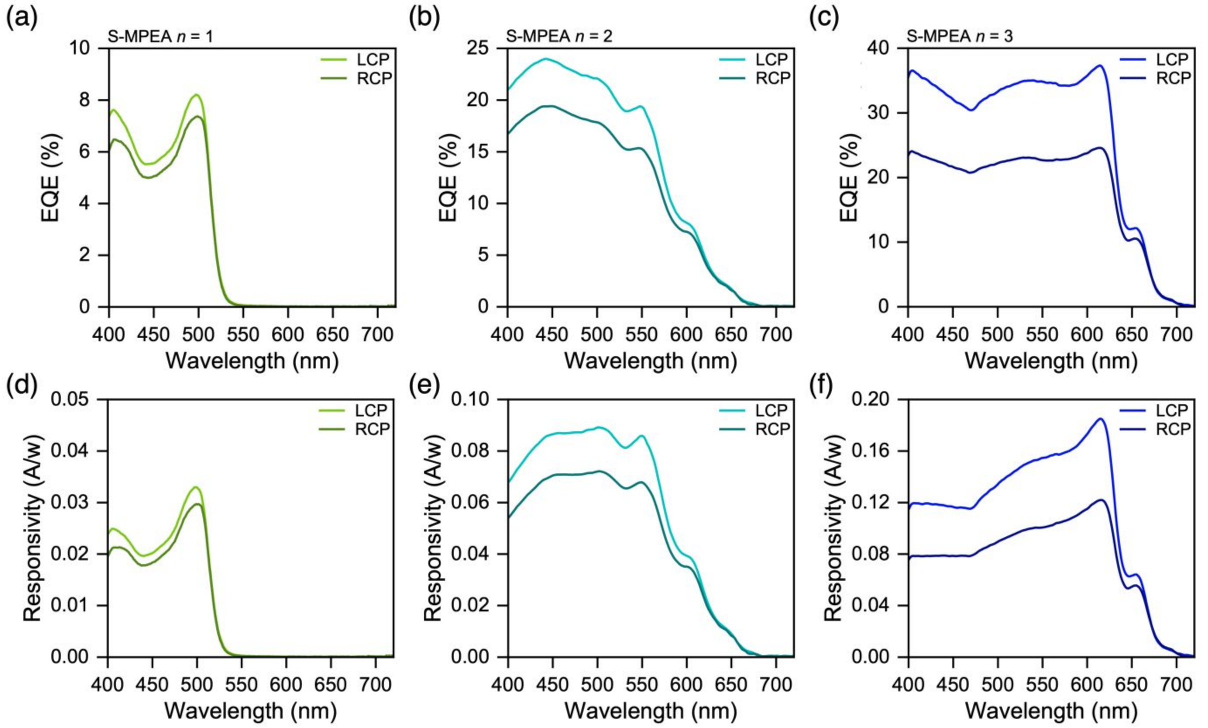

Figure S18. EQE spectra comparing the quantum efficiency (a-c) and responsivity (d-f) of (S-MPEA)$_2$MA$_{n-1}$Pb$_n$I$_{3n+1}$ CPL detectors under LCP and RCP photoexcitation with n = 1 (a, d), n = 2 (b, e) and n = 3 (c, f).

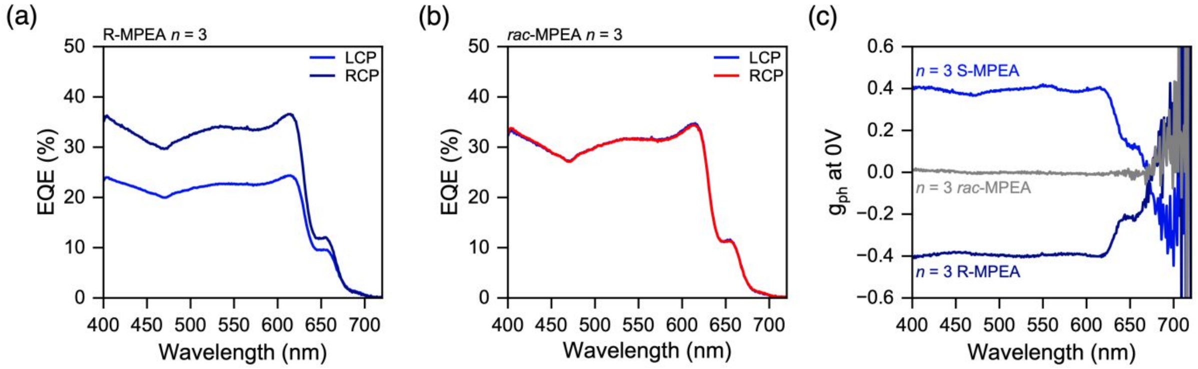

Figure S19. CP-EQE measurements of *n* = 3 (a) $(R\text{-}MPEA)_2MA_2Pb_3I_{11}$ and (b) racemic $(\textit{rac}\text{-}MPEA)_2MA_2Pb_3I_{11}$. (c) Comparison of the spectral evolution of zero-bias g_{ph} of *n* = 3 (R-/ S-/ rac-$MPEA)_2MA_2Pb_3I_{11}$.

Table S2. Summary of anisotropy of circular dichroism (g_{CD}) values for different structurally chiral perovskite compositions.

Material	Dimensionality	g_{CD}	Reference
(R/S-BPEA)$_2$PbI$_4$	2D	1.8 x 10^{-3}	[5]
(R/S-3AMP)PbBr$_4$	2D	1.8 x 10^{-3}	[6]
(R/S-MBA)$_2$PbI$_4$	2D	2.5 x 10^{-3}	[7]
(R/S-MBA)$_2$PbI$_4$	2D	5.7 x 10^{-3}	[8]
(R/S-2-NEA)$_2$PbI$_4$	2D	2.0 x 10^{-3}	[9]
(R/S-MPEA)$_2$EAPb$_2$Br$_7$	Quasi-2D	3.7 x 10^{-4}	[10]
(R/S-MPEA)$_2$CsPb$_2$Br$_7$	Quasi-2D	6.0 x 10^{-4}	[11]
(R/S-MPEA)$_2$Cs$_2$Pb$_3$Br$_{10}$	Quasi-2D	0.7 x 10^{-4}	[11]
(R/S-MBA)$_2$MAPb$_2$Br$_7$	Quasi-2D	7.5 x 10^{-4}	[10]
(R/S-PEA)PbI$_3$	1D	2 x 10^{-2}	[12]
(R/S-NEA)PbI$_3$	1D	4 x 10^{-2}	[13]
**(R/S-MPEA)$_2$PbI$_4$**	**2D**	**2.0 x 10^{-4}**	**This work**
**(R/S-MPEA)$_2$MAPb$_2$I$_7$**	**Quasi-2D**	**8.3 x 10^{-3}**	**This work**
**(R/S-MPEA)$_2$MA$_2$Pb$_3$I$_{10}$**	**Quasi-2D**	**1.7 x 10^{-4}**	**This work**

Table S3. Summary of spin-lifetimes acquired via optical pump-optical probe spectroscopy at room-temperature on different perovskite compositions.

Material	Dimensionality	Chirality	Spin Lifetime (ps)	Reference
$CsPbBr_3$	3D	Achiral	3.7	[14]
$CsPbI_3$	3D	Achiral	4.0	[14]
$MAPbBr_3$	3D	Achiral	4.3	[14]
$CsSnBr_3$	3D	Achiral	2.5	[15]
$MAPbI_3$	3D	Achiral	2.2	[16]
$CsPb(I_{0.1}Br_{0.9})_3$	3D	Achiral	10.0	[17]
$(PEA)_2PbI_4$	2D	Achiral	0.3	[18]
$(BA)_2PbI_4$	2D	Achiral	0.4	[19]
$(BZA)_2PbI_4$	2D	Achiral	0.6	[19]
$(FPEA)_2PbI_4$	2D	Achiral	0.3	[19]
$(PIA)_2PbI_4$	2D	Achiral	7.5	[19]
$(BIA)_2PbI_4$	2D	Achiral	3.3	[19]
$(EOA)_2PbI_4$	2D	Achiral	26.0	[19]
$(BDA)PbI_4$	2D	Achiral	4.3	[20]
$(ODA)PbI_4$	2D	Achiral	2.3	[20]
$(3AMP)PbI_4$	2D	Achiral	10.0	[21]
$(4AMP)PbI_4$	2D	Achiral	24.0	[22]
$(PEA)_2MAPb_2I_7$	Quasi-2D	Achiral	2.9	[18]
$(PEA)_2MA_2Pb_3I_{10}$	Quasi-2D	Achiral	3.1	[18]
$(PEA)_2MA_3Pb_4I_{13}$	Quasi-2D	Achiral	7.0	[18]
$(PEA)_2MASn_2I_7$	Quasi-2D	Achiral	2.9	[23]
$(3AMP)MAPb_2I_7$	Quasi-2D	Achiral	8.0	[21]
$(3AMP)MA_2Pb_3I_{10}$	Quasi-2D	Achiral	7.0	[21]
$(3AMP)MA_3Pb_4I_{13}$	Quasi-2D	Achiral	6.0	[21]
$(4AMP)MAPb_2I_7$	Quasi-2D	Achiral	8.4	[22]
$(4AMP)MA_2Pb_3I_{10}$	Quasi-2D	Achiral	8.4	[22]
**$(R/S\text{-}MPEA)PbI_4$**	**2D**	**Chiral**	**5.0**	**This work**
**$(R/S\text{-}MPEA)_2MAPb_2I_7$**	**Quasi-2D**	**Chiral**	**11.0**	**This work**
**$(R/S\text{-}MPEA)_2MA_2Pb_3I_{10}$**	**Quasi-2D**	**Chiral**	**15.0**	**This work**

Supplementary Note 3. Modelling CPL-detector distinguishability

We modelled g_{ph} as a function of the g_{CD}, spin lifetime and spin-polarized carrier mobilities under different spin-orientations (brought about by different CPL excitation). To develop a correlation between the performance modelling, TRCD measurements and actual devices develop in our work, we limit this analysis under device operation under self-powering conditions (ie. illumination under short-circuit condition) to rule any contribution from variations in built-in electric field brought about by carrier injection. The following assumptions are made:

1. **Zero-bias:** under self-powered conditions, the current is driven purely by the built-in electric field E (imposed by the work functions of the metal electrodes and transport layers). Internal fields or carrier diffusion lengths will drive carrier extraction.
2. **Photocurrent generation**: Photocurrent is assumed to be proportional to the population of carriers generated and successfully extracted. Therefore, all absorbed photons contribute linearly to the photocurrent.
3. **Carrier collection**: Carrier extraction is dominated by drift, as diffusion is negligible under the short-circuit conditions. Thus, the collection efficiency depends on the carrier lifetime and mobility.
4. **1D planar geometry**: The built-in electric field is constant throughout the cross-section of the photodetector, and the generation profile is uniform throughout the device (ie. every point in the stack generates carriers at the same rate). The latter is reasonable given the absorption coefficient and thickness of standard perovskite films.
5. **Nature of carrier recombination**: We model the recombination in the stack as a function of the bulk recombination time τ as the major pathway for carrier recombination in the stack, independent of terms associated with surface or non-radiative recombination in the active layer.
6. **LCP versus RCP**: Based on our experimental setup, the incoming flux of photons under LCP and RCP illumination is indeed the same. Also, the built-in electric field is assumed to be independent of the chirality of the perovskite, same under both LCP and RCP and purely dependent on the choice of transport layers and electrodes used. More critically, the Rashba splitting in the CBM of the CMHS should result in spin-lifetime and mobilities under one polarization (e.g. LCP in case of S-MPEA-materials in our study) to exceed that under RCP, via chirality-induced spin selectivity.

Based on 1, 2 and 3, the photocurrent I is proportional to the absorbed photon flux G and the collection efficiency η_{coll}. That is,

$$I \propto G.\eta_{coll}$$

Absorbed photon flux G (carriers/cm^3.s) at a point x in a film can be represented as a function of the incident photon flux Φ_0 (photons/cm^2.s), the absorption coefficient α (cm^{-1}) and x, the depth into the film in nanometers.

$$G(x) = \Phi_0.\alpha.e^{-\alpha.x}$$

Based on 4, $G(x)$ can be integrated over a film's thickness L (nm) as follows:

$$G = \int_0^L \Phi_0.\alpha.e^{-\alpha.x}$$

$$G = \Phi_0.(1 - e^{-\alpha.L})$$

The product of the absorption coefficient and film thickness yields the absorbance of a film.

$$A = log_{10}(\frac{I_0}{I})$$

We express absorbance A in terms of optical density obtained from standard spectrophotometers as above, where I_0 and I are the incident light intensity entering the sample, and the transmitted light intensity exiting the sample.

$$A = log_{10}(\frac{I_0}{I_0.e^{-\alpha.L}})$$

$$A = log_{10}(e^{\alpha.L})$$

$$A = log_{10}(e^{\alpha.L})$$

$$e^{\alpha.L} = 10^A$$

Thus:

$$G = \Phi_0.(1 - 10^{-A})$$

The collection efficiency was modelled based on the Hecht Equation, which has been used to model charge collection under varying electric fields.[24]

$$\eta_{coll} = \frac{\mu.\tau.V}{L^2}.[1 - e^{\frac{-L^2}{\mu.\tau.V}}]$$

μ refers to the carrier mobility, τ the carrier lifetime, V the applied voltage and L, the active layer thickness. Under short-circuit condition, there is zero-applied voltage. Based on 1 and 3, this equation is rewritten as:

$$\eta_{coll} = \frac{\mu.\tau.E}{L^2}.[1 - e^{\frac{-L}{\mu.\tau.E}}]$$

In the context of chiral perovskites and discriminating photocurrents generated by circularly polarized light for a fixed enantiomer, several of the above parameters exhibit a polarization dependence depending on the extent of selective CPL absorption, band splitting and disparity in effective masses. For instance, due to differential absorption of CPL, A is rewritten as A_{LCP} and A_{RCP}, which are the optical density values at a specific wavelength under left- and right-circularly polarized light respectively. These absorption terms can be correlated with the absorption under unpolarized light and g_{CD} as follows:

$$A = \frac{A_{LCP} + A_{RCP}}{2}$$

$$\Delta A = A_{LCP} - A_{RCP}$$

$$\Delta A\ (optical\ density) = \frac{CD\ (mdeg)}{32980}$$

$$g_{CD} = \frac{CD}{32980.A}$$

$$A_{LCP} = A.(1 + \frac{g_{CD}}{2})$$

$$A_{RCP} = A.(1 - \frac{g_{CD}}{2})$$

Therefore, the polarization dependence of the carrier generation terms can be expressed as below. Note that the photon density Φ_0 is constant for LCP and RCP.

$$\frac{G_{LCP}}{G_{RCP}} = \frac{(1-10^{-A.(1+\frac{g_{CD}}{2})})}{(1-10^{-A.(1-\frac{g_{CD}}{2})})}$$

Similarly, the polarization collection terms are denoted using LCP or RCP in the subscripts. The active thickness L and built-in electric field E (as shown by the J-V results in Figure 5d-f) remains the same regardless of polarization. For CPL detection, preserving spin is critical. After spin-dephasing, the mobility and lifetime are similar to those of carriers generated under unpolarized light. Therefore, the collection efficiency is modelled as a function of carrier mobility (while spin-polarized) and spin-lifetime.

$$\frac{\eta_{LCP}}{\eta_{RCP}} = \frac{\mu_{LCP}.\tau_{LCP}^{S}}{\mu_{RCP}.\tau_{RCP}^{S}}.\frac{[1-e^{\frac{-L}{\mu_{LCP}.\tau_{LCP}^{S}.E}}]}{[1-e^{\frac{-L}{\mu_{RCP}.\tau_{RCP}^{S}.E}}]}$$

Similarly, the polarization dependence of the ratio of photocurrents I generated by LCP and RCP can be represented as:

$$I = \frac{I_{LCP}}{I_{RCP}} = \frac{(1-10^{-A.(1+\frac{g_{CD}}{2})})}{(1-10^{-A.(1-\frac{g_{CD}}{2})})}.\frac{\mu_{LCP}.\tau_{LCP}^{S}}{\mu_{RCP}.\tau_{RCP}^{S}}.\frac{[1-e^{\frac{-L}{\mu_{LCP}.\tau_{LCP}^{S}.E}}]}{[1-e^{\frac{-L}{\mu_{RCP}.\tau_{RCP}^{S}.E}}]}$$

Finally, the anisotropy of spin-polarized currents g_{ph} is:

$$g_{ph} = \frac{2.(I_{LCP}-I_{RCP})}{(I_{LCP}+I_{RCP})} = 2.\frac{(I-1)}{(I+1)}$$

For ease of modelling, the film thickness is kept fixed at 250 nm, according to the cross-section SEM results, τ_{RCP}^{s} at 15 ps and μ_{RCP} at 11.5 cm^2/Vs (as obtained via TRCD and OPTP for the *n* = 3 devices). The ratio of the mobility and spin-lifetime terms are varied by treating the respective terms under LCP illumination as larger. The optical density is assumed to be 1 OD under unpolarized light, to make our calculations more accessible to general conditions.

Additionally, we modelled the polarization dependence of the ratio of photocurrents generated by LCP and RCP in a lateral photoconductor architecture as I_{PC}. Critically, the photoconductive gain must be accounted for under the influence of bias. Photoconductive gain occurs when the photocurrent is amplified by a factor G, such that the ratio of electrons extracted to photons input exceeds 100%.[24] The photoconductive gain G^P is represented by:

$$G^P = \frac{\tau_r}{\tau_t} = \frac{\mu.V.\tau_r}{L^2}$$

Here, τ_r represents the trap-assisted recombination lifetime (non-radiative recombination), while τ_t, the transit time, denotes how fast carriers traverse a channel of length L under a bias V. To account for photoconductive gain, the collection term must now be multiplied by G^P. Note that the μ term in G^P is the only one that demonstrates CPL sensitivity. To the best of our knowledge, spin-selective trapping has not been reported to be a bottleneck in chiral metal halide perovskites. Thus, we obtain:

$$I_{PC} = \frac{I_{LCP}.G_{LCP}^{P}}{I_{RCP}.G_{RCP}^{P}} = \left[\frac{(1-10^{-A.(1+\frac{g_{CD}}{2})})}{(1-10^{-A.(1-\frac{g_{CD}}{2})})}\right].\left[\frac{{\mu_{LCP}}^2.\tau_{LCP}^{S}}{{\mu_{RCP}}^2.\tau_{RCP}^{S}}.\frac{[1-e^{\frac{-L}{\mu_{LCP}.\tau_{LCP}^{S}.V}}]}{[1-e^{\frac{-L}{\mu_{RCP}.\tau_{RCP}^{S}.V}}]}\right]$$

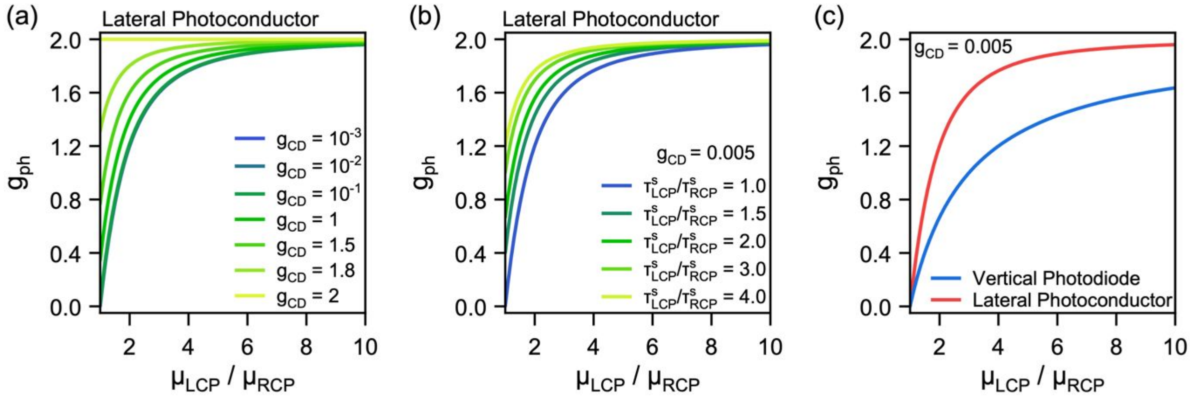

Figure S20. Evolution of g_{ph} as a function of (a) mobility ratio and g_{CD} and (b) mobility ratio and spin-lifetime ratio under different circularly polarized light sources in CMHS photoconductor devices. (c) Comparison of g_{ph} scaling with mobility ratio between photodiode (PD) and photoconductor (PC) architectures.

Based on this analysis, we anticipate that, in principle, chiral photodetectors may elicit superior CPL detection in a lateral photoconductor architecture as the CPL detection has a quadratic dependence on the polarization-dependent carrier mobility term. This trend is represented in **Figure S20a-c**. The gain in g_{ph} with even small changes in mobility differences (**Figure S20a**) of spin-polarized carriers is magnified significantly, while spin-lifetime still retains a significant footprint on g_{ph} (**Figure S20b**). Additionally, charge transport in photoconductors occurs along the lateral direction, with fluent carrier mobility in CMHS. Therefore, in principle, lateral photoconductors structures could enable outstanding CPL discrimination in CMHS (**Figure S20c**).[25] Indeed, CPL photoconductors are more widespread and often have better performance, while CPL photodiodes with $g_{ph} > 0.5$ at 0V are surprisingly sparse.[26, 8]

We also caution that typical CMHS photoconductor devices require large bias to achieve noticeable photodetection (>2 V). As a result, the mobility and spin-lifetime of spin-polarized carriers under different CPL excitation may now exhibit a bias-dependence, which may or may not be detrimental to spin-properties. In addition, the ion-migration effect may become more pronounced at higher voltages, obscuring the influence of spin-properties. This is the rationale behind our selection of photodiodes to correlate CPL detection in a regime where the spin transport will not be affected by additional parameters (i.e. 0 V) and field-induced band bending.

References

(1) Ramakrishnan, S.; Song, D.; Xu, Y.; Zhang, X.; Aksoy, G.; Cotlet, M.; Li, M.; Zhang, Y.; Yu, Q. Solvent-Mediated Formation of Quasi-2D Dion-Jacobson Phases on 3D Perovskites for Inverted Solar Cells Over 23% Efficiency. *Adv. Energy Mater.* **2023**, *13,* 2302240. DOI: 10.1002/aenm.202302240.

(2) Hou, J.; Li, W.; Zhang, H.; Sidhik, S.; Fletcher, J.; Metcalf, I.; Anantharaman, S. B.; Shuai, X.; Mishra, A.; Blancon, J. C.; Katan, C.; Jariwala, D.; Even, J.; Kanatzidis, M. G.; Mohite, A. D. Synthesis of 2D Perovskite Crystals via Progressive Transformation of Quantum Well Thickness. *Nat. Synth.* **2024**, *3,* 265–275. DOI: 10.1038/s44160-023-00422-3.

(3) S. Ramakrishnan, B. Chen, X. Zhang, Y. Xie, X. Tong, Y. Xu, A. N. Alphenaar, A. Ruthen, A. J. Babatunde, Y. Zhang, M. Cotlet, D. B. Mitzi, Q. Yu. Phase-Stabilized 2D/3D Hetero-Bilayers via Lattice Matching for Efficient and Stable Inverted Solar Cells. *Joule.* **2025**, *9*, 101954. DOI: 10.1016/j.joule.2025.101954.

(4) Ramakrishnan, S.; Li, H.; Xu, Y.; Shin, D.; Dursun, I.; Cotlet, M.; Zhang, Y.; Yu, Q. Ruddlesden – Popper Perovskites with Narrow Phase Distribution for Air-Stable Solar Cells. **2022**, *Sol. RRL, 6,* 2200490. DOI: 10.1002/solr.202200490.

(5) Peng, Y.; Liu, X.; Li, L.; Yao, Y.; Ye, H.; Shang, X.; Chen, X.; Luo, J. Realization of Vis-NIR Dual-Modal Circularly Polarized Light Detection in Chiral Perovskite Bulk Crystals. *J. Am. Chem. Soc.* **2021**, *143*, 14077–14082. DOI: 10.1021/jacs.1c07183.

(6) Fan, C. C.; Han, X. Bin; Liang, B. D.; Shi, C.; Miao, L. P.; Chai, C. Y.; Liu, C. D.; Ye, Q.; Zhang, W. Chiral Rashba Ferroelectrics for Circularly Polarized Light Detection. *Adv. Mater.* **2022**, *34,* 2204119. DOI: 10.1002/adma.202204119.

(7) Scalon, L.; Brunner, J.; Guaita, M. G. D.; Szostak, R.; Albaladejo-Siguan, M.; Kodalle, T.; Guerrero-León, L. A.; Sutter-Fella, C. M.; Oliveira, C. C.; Vaynzof, Y.; Nogueira, A. F. Tuning Phase Purity in Chiral 2D Perovskites. *Adv Opt Mater* **2024**, *12,* 2300776. DOI: 10.1002/adom.202300776.

(8) H. Kim, W. Choi, Y. J. Kim, J. Kim, J. Ahn, I. Song, M. Kwak, J. Kim, J. Park, D. Yoo, J. Park, S. K. Kwak. Giant Chiral Amplification of Chiral 2D Perovskites via Dynamic Crystal Reconstruction. *Sci. Adv.*, **2024**. *10*, 34, DOI: 10.1126/sciadv.ado5942.

(9) Son, J.; Ma, S.; Jung, Y. K.; Tan, J.; Jang, G.; Lee, H.; Lee, C. U.; Lee, J.; Moon, S.; Jeong, W.; Walsh, A.; Moon, J. Unraveling Chirality Transfer Mechanism by Structural Isomer-Derived Hydrogen Bonding Interaction in 2D Chiral Perovskite. *Nat. Commun.* **2023**, *14*, 3214. DOI: 10.1038/s41467-023-38927-2.

(10) Wu, W.; Shang, X.; Xu, Z.; Ye, H.; Yao, Y.; Chen, X.; Hong, M.; Luo, J.; Li, L. Toward Efficient Two-Photon Circularly Polarized Light Detection through Cooperative Strategies in Chiral Quasi-2D Perovskites. *Adv. Sci.* **2023**, *10*, 2206070. DOI: 10.1002/advs.202206070.

(11) Cao, Q.; Song, R.; Chan, C. C. S.; Wang, Z.; Wong, P. Y.; Wong, K. S.; Blum, V.; Lu, H. Chiral Perovskite Nanoplatelets with Tunable Circularly Polarized Luminescence in the Strong Confinement Regime. *Adv Opt Mater* **2023**, *11*, 2203125. DOI: 10.1002/adom.202203125.

(12) Chen, C.; Gao, L.; Gao, W.; Ge, C.; Du, X.; Li, Z.; Yang, Y.; Niu, G.; Tang, J. Circularly Polarized Light Detection Using Chiral Hybrid Perovskite. *Nat. Commun.* **2019**, *10*, 1927. DOI: 10.1038/s41467-019-09942-z.

(13) A. Ishii, T. Miyasaka. Direct Detection of Circular Polarized Light in Helical 1D Perovskite-Based Photodiode. *Sci. Adv.* **2020**, 6, 46, eabd3274 .DOI:10.1126/sciadv.abd3274.

(14) Zhou, M.; Sarmiento, J. S.; Fei, C.; Zhang, X.; Wang, H. *J.* Effect of Composition on the Spin Relaxation of Lead Halide Perovskites. *J. Phys. Chem. Lett.* **2020**, *11*, 1502–1507. DOI: 10.1021/acs.jpclett.0c00004.

(15) Xu, J.; Li, K.; Huynh, U. N.; Fadel, M.; Huang, J.; Sundararaman, R.; Vardeny, V.; Ping, Y. How Spin Relaxes and Dephases in Bulk Halide Perovskites. *Nat Commun* **2024**, *15*, 187. DOI: 10.1038/s41467-023-42835-w.

(16) Giovanni, D.; Ma, H.; Chua, J.; Grätzel, M.; Ramesh, R.; Mhaisalkar, S.; Mathews, N.; Sum, T. C. Highly Spin-Polarized Carrier Dynamics and Ultralarge Photoinduced Magnetization in CH3NH3PbI3 Perovskite Thin Films. *Nano. Lett.* **2015**, *15*, 1553–1558. DOI: 10.1021/nl5039314.

(17) Y.-H. Kim, Y. Zhai, H. Lu, X. Pan, C. Xiao, E. A. Gaulding, S. P. Harvey, J. J. Berry, Z. Valy Vardeny, J. M. Luther, M. C. Beard. Chiral-Induced Spin Selectivity Enables a Room-Temperature Spin Light-Emitting Diode. *Science*. **2021**. *371*, 1129-1133. DOI: 10.1126/science.abf5291.

(18) Chen, X.; Lu, H.; Li, Z.; Zhai, Y.; Ndione, P. F.; Berry, J. J.; Zhu, K.; Yang, Y.; Beard, M. C. Impact of Layer Thickness on the Charge Carrier and Spin Coherence Lifetime in Two-Dimensional Layered Perovskite Single Crystals. *ACS. Energy Lett.* **2018**, *3*, 2273–2279. DOI: 10.1021/acsenergylett.8b01315.

(19) Chen, X.; Lu, H.; Wang, K.; Zhai, Y.; Lunin, V.; Sercel, P. C.; Beard, M. C. Tuning Spin-Polarized Lifetime in Two-Dimensional Metal-Halide Perovskite through Exciton Binding Energy. *J. Am. Chem. Soc.* **2021**, *143*, 19438–19445. DOI: 10.1021/jacs.1c08514.

(20) Lei, H.; Xu, Y.; Zhang, Y.; Feng, Q.; Zhou, H.; Tang, W.; Wang, J.; Li, L.; Nan, G.; Xu, W.; Zhu, H. Persistent Exciton Dressed by Weak Polaronic Effect in Rigid and Harmonic Lattice Dion-Jacobson 2D Perovskite. *ACS Nano.* **2024**, *18,* 31485–31494. DOI: 10.1021/acsnano.4c12132.

(21) Huang, Y.; Chen, C.; Gong, S.; Hu, Q.; Liu, J.; Chen, H.; Mao, L.; Chen, X. Tuning Spin-Polarized Lifetime at High Carrier Density through Deformation Potential in Dion-Jacobson-Phase Perovskites. *J. Am. Chem. Soc.* **2024**, *146*, 12225–12232. DOI: 10.1021/jacs.4c03532.

(22) Huang, Y.; Gong, S.; Chen, Q.; Chen, C.; Yang, Z.; Wang, K.; Xue, J.; Wang, D.; Lu, H.; Mao, L.; Yang, Y.; Zhao, J.-Z.; Chen, X. Giant Deformation Potential Induced Small Polaron Effect in Dion–Jacobson Two-Dimensional Lead Halide Perovskites. *Natl. Sc.i Rev.* **2025**, *12*, nwae461, DOI: 10.1093/nsr/nwae461.

(23) Zhou, H.; Feng, Q.; Sun, C.; Li, Y.; Tao, W.; Tang, W.; Li, L.; Shi, E.; Nan, G.; Zhu, H. Robust Excitonic Light Emission in 2D Tin Halide Perovskites by Weak Excited State Polaronic Effect. *Nat. Commun.* **2024**, *15,* 8541. DOI: 10.1038/s41467-024-52952-9.

(24) K. R. Dudipala, T.-H. Le, W. Nie, R. L. Z. Hoye, Halide Perovskites and Their Derivatives for Efficient, High-Resolution Direct Radiation Detection: Design Strategies and Applications. *Adv. Mater.* **2024**, 36, 2304523. DOI: 10.1002/adma.202304523.

(25) L. Wang, W. Hao, B. Peng, J. Ren, H. Li, Nucleation-Controlled Crystallization of Chiral 2D Perovskite Single Crystal Thin Films for High-Sensitivity Circularly Polarized Light Detection. *Adv. Mater.* 2025, 37, 2414199. DOI: 10.1002/adma.202414199.

(26) X. Zhang, Y. Xu, A. N. Alphenaar, S. Ramakrishnan, Y. Zhang, A. J. Babatunde, Q. Yu*, Self-Powered Circularly Polarized Light Detection Enabled by Chiral Two-Dimensional Perovskites with Mixed Chiral–Achiral Organic Cations. *ACS Nano* 2024, 18, 22, 14605–14616. DOI: 10.1021/acsnano.4c02588.
